# Cyanophenylalanine as an Infrared Probe for Iron–Sulfur Cluster Redox State in Multicenter Metalloenzymes

**DOI:** 10.1002/cbic.202500251

**Published:** 2025-05-26

**Authors:** Zehui Duan, Jiaao Wei, Stephen B. Carr, Miguel Ramirez, Rhiannon M. Evans, Philip A. Ash, Patricia Rodriguez‐Macia, Amit Sachdeva, Kylie A. Vincent

**Affiliations:** ^1^ Department of Chemistry University of Oxford Inorganic Chemistry Laboratory, South Parks Road Oxford OX1 3QR UK; ^2^ Research Complex at Harwell Rutherford Appleton Laboratory Harwell Campus Didcot OX11 0FA UK; ^3^ School of Chemistry University of East Anglia Norwich Research Park Norwich NR4 7TJ UK; ^4^ Present address: School of Chemistry and Leicester Institute for Structural and Chemical Biology University of Leicester University Road Leicester LE1 7RH UK

**Keywords:** cyanophenylalanine, hydrogenase, infrared labels, iron–sulfur clusters, spectroelectrochemistry

## Abstract

The noncanonical amino acid, para‐cyanophenylalanine (CNF), when incorporated into metalloproteins, functions as an infrared spectroscopic probe for the redox state of iron‐sulfur clusters, offering a strategy for determining electron occupancy in the electron transport chains of complex metalloenzymes. A redshift of ≈1–2 cm^−1^ in the nitrile (NC) stretching frequency is observed, following reduction of spinach ferredoxin modified to contain CNF close to its [2Fe–2S] center, and this shift is reversed on re‐oxidation. We extend this to CNF positioned near to the proximal [4Fe–4S] cluster of the [FeFe] hydrogenase from *Desulfovibrio desulfuricans*. In combination with a distal [4Fe–4S] cluster and the [4Fe–4S] cluster of the active site ‘H‐cluster’ ([4Fe–4S]_H_), the proximal cluster forms an electron relay connecting the active site to the surface of the protein. Again, a reversible shift in wavenumber for CNF is observed, following cluster reduction in either apo‐protein (containing the iron‐sulfur clusters but lacking the active site) or holo‐protein with intact active site, demonstrating the general applicability of this approach to studying complex metalloenzymes.

## Graphical abstract

The noncanonical amino acid, cyanophenylalanine, is genetically inserted close to iron–sulfur clusters of either spinach ferredoxin or iron–iron hydrogenase, and functions as an infrared spectroscopic reporter for changes in cluster redox state.

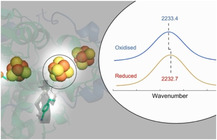

## 1 Introduction

Iron–sulfur (FeS) enzymes catalyze key activation processes of small molecules, including the cycling of H^+^/H_2_ in hydrogenases, the interconversion of CO_2_/CO in carbon monoxide dehydrogenases, and N_2_ reduction to ammonia in nitrogenases. Much attention has been paid to redox changes at the active sites of these enzymes during catalysis.^[^ ^1^ ^]^ In hydrogenases, this has been facilitated by strong infrared (IR) absorption bands from the endogenous CO and CN^−^ ligands located at their active sites, which are highly sensitive to the redox state of the active‐site cluster. These have been exploited to examine the redox chemistry of hydrogenase active sites in solution, during catalytic turnover on an electrode and in single crystals.^[^ ^2^ ^]^ However, it is becoming increasingly apparent that long‐range effects in the electron relay clusters of multicenter enzymes also affect their catalytic properties.^[^ ^3^ ^]^ The [NiFe] hydrogenases typically have an electron‐relay chain of three FeS clusters, while the relay chain of [FeFe] hydrogenases varies from a single to multiple FeS clusters. A spacing of ≈10–14 Å between redox centers enables fast electron tunneling to keep pace with high rates of catalytic turnover, even when individual electron transfer steps within the relay chain may be energetically unfavorable.^[^ ^4^ ^]^ Although different types of FeS clusters exhibit characteristic electron paramagnetic resonance (EPR) signals in their paramagnetic states, it is often difficult to assign spectral contributions to specific clusters in a multicenter protein.^[^ ^5^ ^]^ Since EPR spectra for FeS clusters are typically recorded at cryogenic temperatures, this approach is poorly suited for room‐temperature turnover studies. FeS centers are typically red/brown/black in color, and absorption features in the UV–visible region are broad, weak, and overlapping; consequently, there are also no obvious absorption bands to excite into for resonance Raman spectroscopy. FeS cluster vibrations are weak and occur in the far IR region (≈200–450 cm^−1^), and although some redox state‐dependent changes have been observed in this frequency range for simple ferredoxins,^[^ ^6^ ^]^ they would be difficult to assign to specific clusters in multicenter enzymes. Changes in FeS vibrations have also been studied by nuclear resonance vibrational spectroscopy (NRVS) aided by density functional theory calculations,^[^ ^7^ ^]^ but NRVS studies are complicated by the requirement for ^57^Fe‐labeled proteins, and again are carried out on frozen samples.

Para‐cyanophenylalanine (CNF) is a noncanonical amino acid that exhibits a characteristic vibrational band in the mid–IR region resulting from the nitrile (NC) stretch of the cyano substituent. It can be incorporated into proteins via an orthogonal, engineered aminoacyl‐tRNA synthetase, and has been used to probe dynamics, local micro‐environments^[^ ^8^ ^]^ and electric field effects^[^ ^9^ ^]^ in proteins. We set out to explore whether CNF incorporated close to a FeS cluster could function as a convenient mid‐IR reporter for the redox state of a specific FeS cluster, in conjunction with electrochemical and chemical modulation of redox states of the protein. We first selected spinach ferredoxin I (FdI) and incorporated CNF close to the [2Fe–2S] cluster. A reproducible shift in nitrile stretching frequency with cluster redox state was observed in the IR spectrum in spinach ferredoxin. This encouraged us to incorporate CNF into a multicenter iron–sulfur enzyme, *Desulfovibrio desulfuricans* [FeFe] hydrogenase (*Dd*HydAB). This enzyme is ideal for this purpose because, in addition to the active site H‐cluster comprising a [2Fe]_H_ catalytic site covalently linked to a [4Fe4S]_H_ cluster, it has two additional [4Fe–4S] clusters, forming an electron relay chain to the protein surface. CNF was incorporated between [4Fe–4S]_H_ of the active site and the [4Fe–4S] cluster ‘proximal’ to the active site, and again the nitrile stretching band shifted reversibly with redox state, confirming its utility as a probe for iron‐sulfur cluster redox chemistry within a larger enzyme.

## 2 Results and Discussion

Spinach FdI was selected as the first model system because of its small size and the accessible redox change in its [2Fe‐2S] cluster. We established a suitable overexpression system for glutathione S‐transferase (GST)‐tagged FdI in *Escherichia coli*.^[^ ^10^ ^]^ Recombinant FdI was purified by GST‐affinity chromatography followed by cleavage of the GST tag, and further enriched by anion‐exchange and size‐exclusion chromatography for crystallization (see Supporting Information Methods). The reduction potential of wild‐type (WT)‐FdI determined by cyclic voltammetry, −0.40 V versus the standard hydrogen electrode (SHE) (all potentials are subsequently quoted vs SHE), and the UV–visible spectra for air‐oxidized and dithionite‐reduced FdI (see Figure S4, Supporting Information) were consistent with previous reports.^[^ ^11^ ^]^

Next, aromatic amino acids close to the FeS cluster in FdI were tested for their plasticity to mutagenesis by conservative alterations to canonical aromatic amino acids. Mutation of Tyr in position 37 (Y37) gave protein variants with equivalent UV–visible spectra and cluster reduction potentials to the WT FdI, while variants at other sites gave abnormal spectral features (Figure S5 and S6, Supporting Information). Therefore, site 37 was selected for site‐specific replacement with CNF, to generate the FdI variant ‘Y37CNF’. To express the FdI‐Y37CNF variant, cells were supplemented with plasmids containing genes for expression of: i) FdI where the codon at position 37 is changed to TAG (amber) stop codon, ii) an engineered CNF‐specific *Methanocaldococcus janaschii* aminoacyl‐tRNA synthetase (*Mj*(CNF)RS), and iii) suppressor *M. janaschii* tRNA (*Mj*tRNA_CUA_) (see Supporting Information methods). The Y37CNF FdI also exhibited a UV–visible spectrum and reduction potential indistinguishable from the WT protein (Figure S6, Supporting Information).

**Figure 1:**
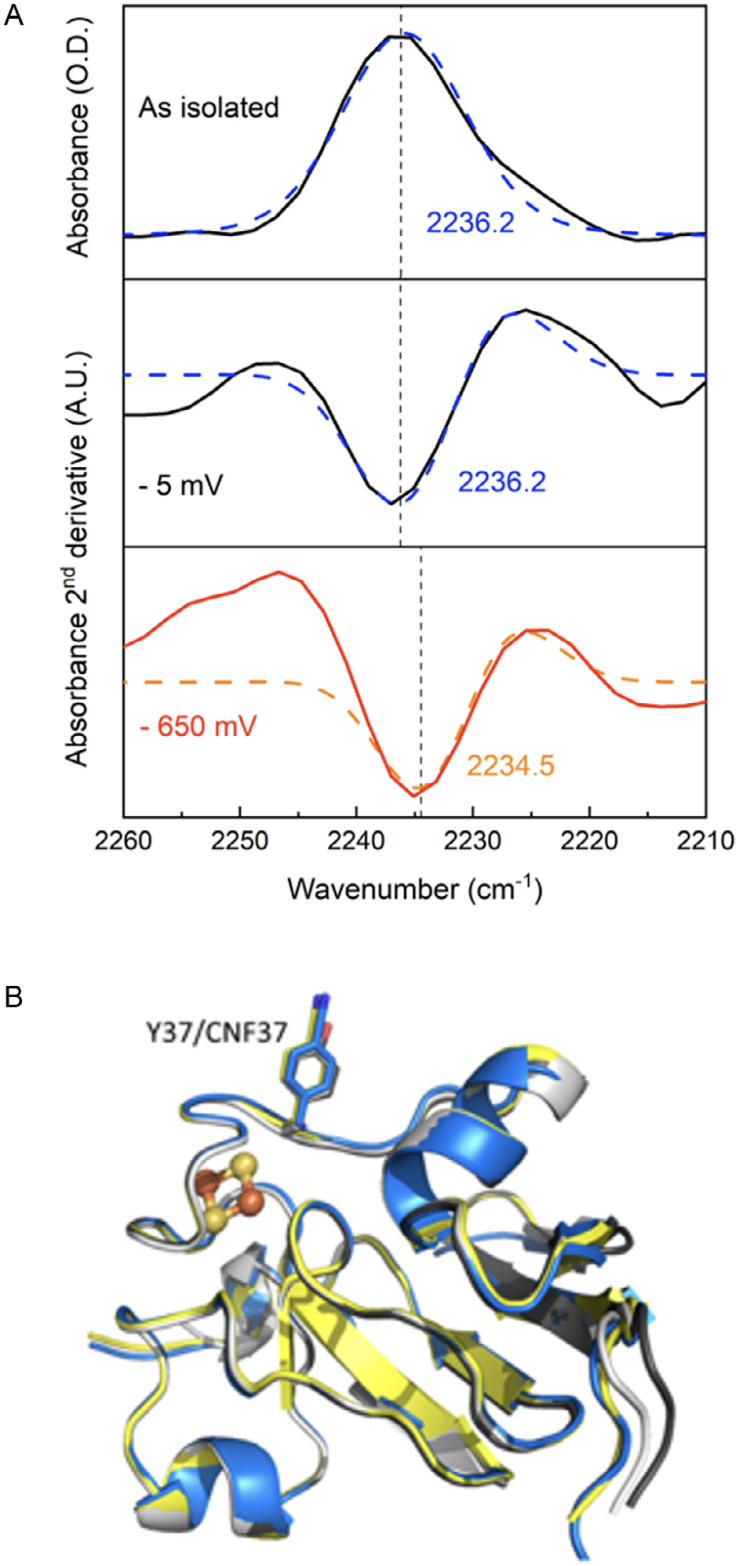
A) IR spectroscopy study of Y37CNF FdI. Top: Transmission‐mode IR spectrum of as‐isolated Y37CNF FdI (dark solid line), recorded against a buffer background and corrected for baseline slope. Peak fitting by Voigt profile (see Supporting Information)^[^ ^21^ ^]^ is shown as a blue dashed line. Conditions: 100 mM Tris‐HCl buffer, pH 8, with 150 mM NaCl, 25 °C, 2 cm^−1^ resolution, and 1024 co‐averaged scans. Middle and bottom: 2nd derivative IR spectra recorded in reflection‐absorption mode at a polished glassy carbon working electrode^[2a]^ for Y37CNF FdI (≈2 mM) at potentials of −5 mV (middle, oxidized) and −650 mV (bottom, reduced), respectively (solid line). The peak positions were determined from peak fitting to a 2nd derivative Gaussian function. Conditions: 100 mM Tris‐HCl buffer, pH 8, with 150 mM NaCl, 25 °C, 2 cm^−1^ resolution, and 1024 co‐averaged scans. B) Overlay of FdI structures solved as part of this study shows that the region around the [2Fe–2S] cluster is unperturbed by incorporation of CNF or by changes in its redox state (r.m.s.d in Cα positions relative to oxidized WT protein shown in brackets): oxidized WT FdI (white), reduced WT FdI (gray, 0.36 Å), oxidized Y37CNF FdI (yellow, 0.47 Å) and reduced Y37CNF FdI (blue, 0.61 Å).

The IR spectra of Y37CNF FdI in the as‐isolated state in solution show a low‐intensity but clear nitrile stretching band at 2236.2 cm^−1^ (**Figure** **1**A, top) close to that of free CNF measured in the same buffer solution (2237 cm^−1^, Figure S8, Supporting Information). We then applied electrochemical control to the solution of Y37CNF FdI in an IR spectro‐electrochemical cell, Figure 1A (middle and bottom). At −5 mV, the spectrum exactly resembled the as‐isolated protein, while at −650 mV, a redshift of 1.7 cm^−1^ was observed in the band from CNF. The shift in NC stretching frequency was reproduced over a second reduction/re‐oxidation sequence on the same sample. Since the CNF is close to the protein surface (see below and Figure 1B), it was important to confirm that this shift did not simply arise from the proximity of the CNF to the charged electrode interface. We therefore performed a similar spectro‐electrochemical experiment with a solution of free CNF amino acid and found no shift when the same potential sequence was applied (Figure S8, Supporting Information). We attempted chemical reduction of Y37CNF FdI using sodium dithionite, but spectra of the reduced protein were unfortunately too low in intensity for clear spectral assignment. The shift of 1.7 cm^−1^ observed for Y37CNF FdI upon electrochemical reduction/ oxidation is comparable in magnitude to the IR shift observed with electrode potential in electrode‐immobilized CNF modified cytochrome‐c, although in this case, the shift was attributed to long‐range effects between the electrified interface and the immobilized protein.^[9b]^

Crystal structures of WT FdI and variant Y37CNF were solved at near‐atomic resolution for both oxidized and reduced states of the protein (Table S3 and S4, Supporting Information). Superposition of the structures (Figure 1B) confirms that the cluster environment is well‐preserved in the CNF‐modified protein, and is unaltered by redox state (see also Figure S3–S5, Supporting Information). Site‐specific replacement of tyrosine with CNF causes a small change in conformation of the side chain relative to the native enzyme. This shift is sufficient to alter the crystal packing, placing the CNF side chain in a slightly different environment within the lattice relative to the native Tyr, however, comparison of the structures of oxidized and reduced Y37CNF FdI demonstrates that the redox state of Fe does not influence the geometry of the side chain relative to the [2Fe–2S] cluster position (Figure 1B). Furthermore, the Y37CNF FdI variant is fully metalated, and analysis of the temperature factors of amino acids surrounding the mutated residue shows that incorporation of the non‐native residue does not alter the flexibility of the protein in this region. Differential scanning fluorimetry data (Table S2, Supporting Information) reveal similar melting temperatures for WT and Y37CNF variant FdI, suggesting the structural similarities are maintained in solution. Together, these observations indicate that the observed change in frequency for the nitrile stretching band following reduction results from differences in the redox state of the [2Fe–2S] cluster rather than a positional change of the CNF probe.

Overall, these measurements provide confidence that the NC vibrational band of CNF is responding to the redox state of the [2Fe–2S] cluster of FdI. This encouraged us to incorporate CNF into a larger, multiredox center enzyme, *Dd*HydAB. In this enzyme, the frequency of vibrational bands from the CO and CN^−^ ligands at the [2Fe] subcluster of the active site is subtly affected by the redox state of the covalently linked [4Fe4S] cluster and even the proximal [4Fe–4S] cluster,^[^ ^12^ ^]^ but the interplay between redox state of the other two clusters and the active site during catalysis is not well understood. The ability to ‘maturate’ apo‐enzymes of the [FeFe] hydrogenase with synthetically‐produced precursors of the [2Fe] site enables good yields of this protein because the apo‐protein (incorporating the iron–sulfur clusters but not the [2Fe] subsite) can be over‐expressed readily in *E. coli*.^[^ ^13^ ^]^ The apo‐protein maturated with [Fe_2_(adt)(CO)_4_(CN)_2_]^2‐^ (adt = azadithiolate) gives holo‐protein (*Dd*HydAB^ADT^) with an identical active site to the natively expressed enzyme,^[^ ^13^ ^]^ whereas maturation with a propanedithiolate (pdt)‐ligated cluster gives a redox‐active but catalytically inactive form of the enzyme, *Dd*HydAB^PDT^.^[13b]^

**Figure 2:**
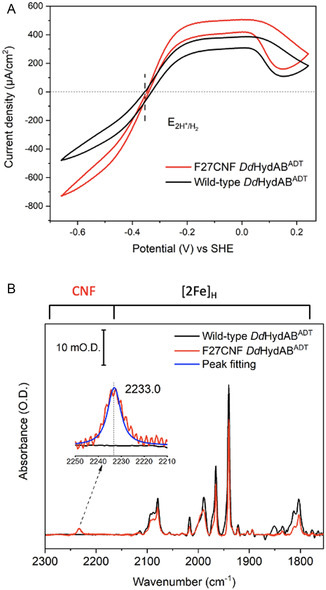
A) Cyclic voltammograms of WT *Dd*HydAB^ADT^ and F27CNF *Dd*HydAB^ADT^, adsorbed onto a pyrolytic graphite electrode. Conditions: buffer mix pH 6, 2000 rpm, 1000 mL min^−1^ H_2_, and scan rate 20 mV s^−1^. B) Transmission‐mode IR spectra WT *Dd*HydAB^ADT^ (1 mM) and F27CNF *Dd*HydAB^ADT^ (1.5 mM). Conditions: 100 mM Tris‐HCl buffer + 150 mM NaCl pH 8, 25 °C, 1 cm^−1^ resolution, and 1024 co‐averaged scans.

**Figure 3:**
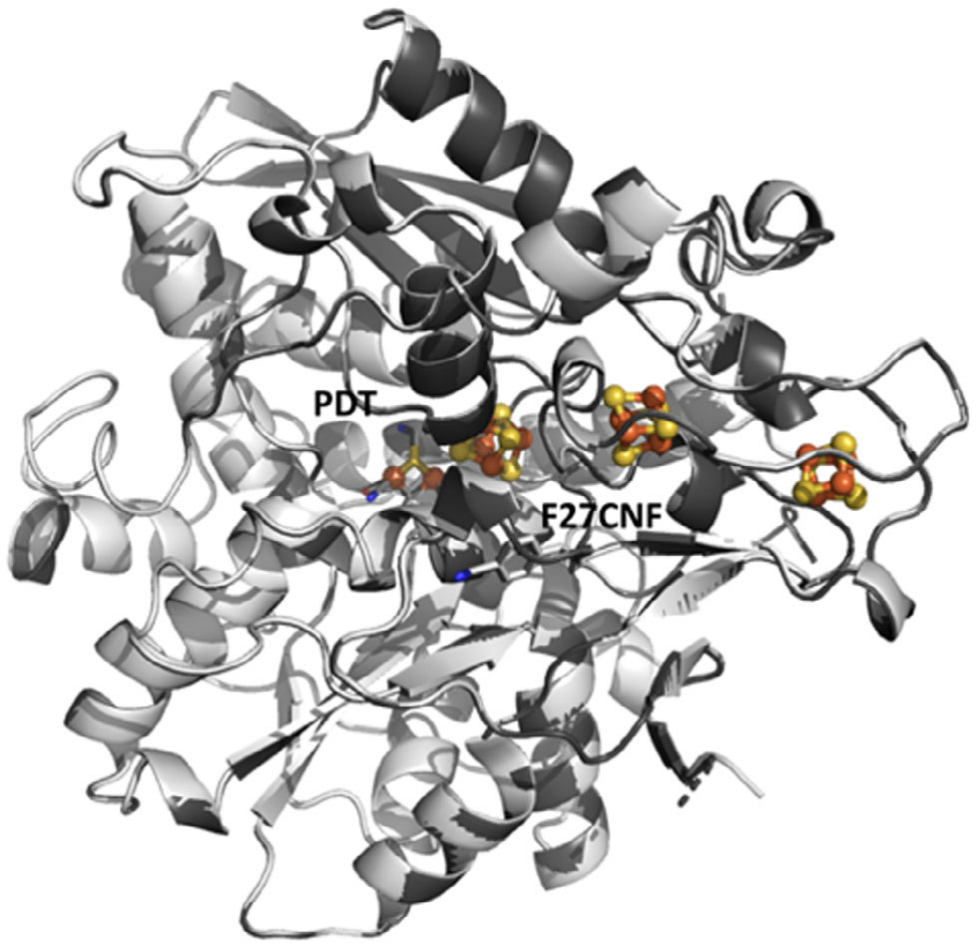
Structures of F27CNF *Dd*HydAB^PDT^ in oxidized (white) and reduced (gray) states show the geometry of the CNF probe is unaltered by redox changes in the protein (r.m.s.d. in Cα positions 0.1 Å). Oxidant: 5 mM potassium ferricyanide; reductant: 5 mM sodium dithionite.

We chose the proximal FeS cluster for study, due to its position in the middle of the electron transport chain, where the CNF probe could be protected from exposure to the solvent or electrode interface. We identified two residues close to the proximal [4Fe–4S] cluster, F27 and F51, in apo‐*Dd*HydAB as possible target residues for mutation to CNF (Figure S10, Supporting Information). However, the efficiency of site‐specific incorporation of CNF at position 51 was poor, so this variant was not taken forward. The F27CNF mutation, on the other hand, resulted in a full‐length protein containing the non‐native cyanophenylalanine amino acid at position 27, termed F27CNF apo‐*Dd*HydAB. When maturated with the adt‐containing precursor, H_2_‐dependent solution activity assays following the reduction of 1 mM benzyl viologen by the enzyme showed that the holo‐enzyme F27CNF *Dd*HydAB^ADT^ is at least as active as the WT protein for H_2_‐oxidation (Figure S15, Supporting Information). Additionally, the shape of the voltammetric response for F27CNF *Dd*HydAB^ADT^ compared to the WT protein when probed by protein film electrochemistry is indistinguishable (**Figure** **2**A). An IR spectrum for F27CNF *Dd*HydAB^ADT^ in the as‐isolated state is shown in Figure 2B (red line). Bands arising from the endogenous CO and CN^−^ ligands of the active site [2Fe]_H_ are identical to those of as‐isolated WT *Dd*HydAB^ADT^ (black line) in the 1750 to 2150 cm^−1^ region, showing an intense band at 1940 cm^−1^ characteristic of the H_ox_ state.^[^ ^14^ ^]^ This further confirms the integrity of the CNF‐modified protein. Additionally, we observe a lower‐intensity but clear band at 2233.0 cm^−1^ in F27CNF *Dd*HydAB^ADT^, absent in WT *Dd*HydAB^ADT^, which we assign to the NC stretching band of the CNF. An intensity for the NC stretch in the low mO.D. range is consistent with studies of CNF in aqueous solution, which give an extinction coefficient around 230 M^−1^ cm^−1^.^[^ ^15^ ^]^ High resolution crystal structures were solved for F27CNF apo‐*Dd*HydAB (Figure S16, Supporting Information), and F27CNF *Dd*HydAB^PDT^ in oxidized and reduced states (**Figure** **3**, see also Table S5, Supporting Information) and show that incorporation of CNF is well tolerated with minimal perturbation of the protein structure or flexibility compared to the WT enzyme (PDB: 6SG2).^[^ ^16^ ^]^ The conformation of the CNF group was also unaffected by manipulation of the redox state of the metal clusters (Figure 3). The nitrile group of CNF lies ≈12 Å from the proximal [4Fe–4S] cluster, and 13 Å from the [4Fe4S]_H_ of the H‐cluster (see Figure 3 and S16C, Supporting Information).

**Figure 4:**
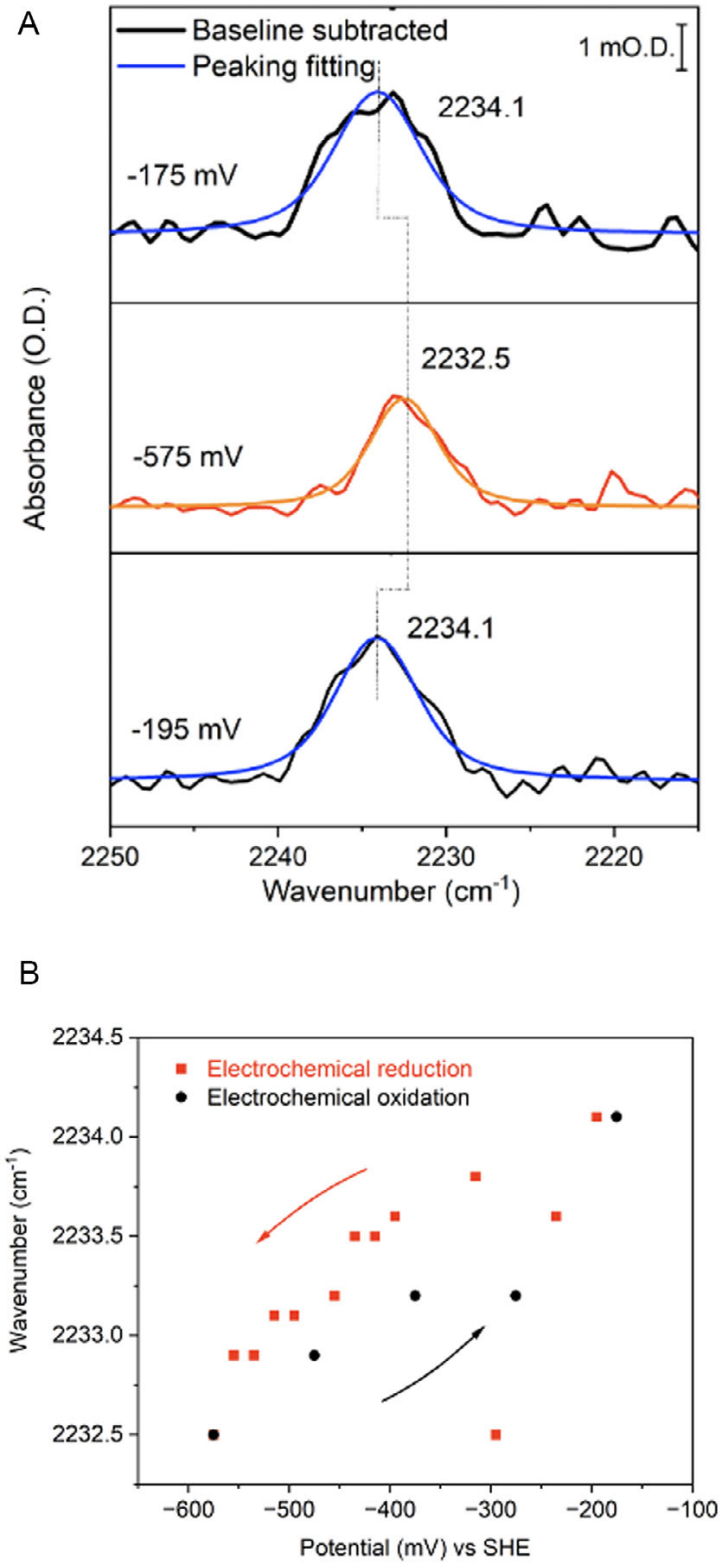
A) IR spectroelectrochemistry of F27CNF apo‐*Dd*HydAB. Spectra were recorded in a reflection‐absorption cell. Conditions: 1.5 mM F27CNF apo‐*Dd*HydAB in 100 mM Tris‐HCl + 150 mM NaCl pH 8 buffer containing redox mediators (see Supporting Information, Experimental Section), with peak fitting according to a Voigt profile; 1 cm^−1^ resolution, and 1024 co‐averaged scans. B) NC stretching band of CNF versus applied potential. The red dots represent titration in a reductive direction, and the black dots represent return titration series in an oxidative direction.

To probe the ability of CNF to sense redox changes in the FeS cluster relay, we first examined F27CNF apo‐*Dd*HydAB in its as‐isolated state (EPR spectroscopy on F27CNF apo‐*Dd*HydAB indicates that the FeS clusters are 85% oxidized in the as‐isolated state, Figure S19, Supporting Information). The nitrile stretching band was easily distinguished at 2233.5 cm^−1^ (Figure S12, Supporting Information). We then subjected the F27CNF apo‐*Dd*HydAB to electrochemically‐controlled redox titration with in situ IR detection (**Figure** **4**). The potential was first poised at −175 mV, and peak fitting situated the peak maximum of the nitrile stretching band of CNF at 2234.1 cm^−1^, suggesting a slight further oxidation under the potential control compared to the as‐isolated protein. The potential was then stepped successively down to −575 mV in 20 mV steps, with spectra recorded after electrochemical equilibration at each potential (Figure 4B). A steady redshift in the wavenumber of the nitrile stretching band was observed over this potential range, down to 2232.5 cm^−1^ at the lowest potential, representing a total shift of 1.6 cm^−1^. This was reversed on re‐oxidation (Figure 4B). This shift is very similar to that reported for CNF‐close to a [4Fe‐4S] cluster in a bacterial ferredoxin (containing two [4Fe–4S] clusters) in complex with the partner [FeFe] hydrogenase I from *Clostridium pasteurianum* when the gas atmosphere was switched between N_2_ to H_2_ to effect reduction of the hydrogenase and electron transfer to the ferredoxin.^[^ ^17^ ^]^

We then explored F27CNF *Dd*HydAB^PDT^, which allowed us to use IR spectroscopy to probe both the redox state of the H‐cluster^PDT^ (via the endogenous CN^−^ and CO ligands) and the iron–sulfur cluster relay (via the CNF band position). The catalytically inactive *Dd*HydAB^PDT^ with a propanedithiolate‐bridged cluster shows similar structural and spectroscopic properties to the active *Dd*HydAB^ADT^, but with simplified redox behavior, which makes it easier to monitor the probe effects of CNF without complications from catalytic proton reduction. Again, the spectrum of F27CNF *Dd*HydAB^PDT^ closely matches that of the WT *Dd*HydAB^PDT^ in the 1750–2150 cm^−1^ region, both featuring the band characteristic of the well‐known H_ox_^PDT^ state.^[13b]^ F27CNF *Dd*HydAB^PDT^ shows an additional band at 2233.5 cm^−1^, corresponding to the nitrile stretch of CNF, very similar to that observed in the apo‐protein. Following a chemical reduction with Eu(II)‐DTPA (DTPA = diethylenetriamine pentaacetate), the H‐cluster^PDT^ bands shift toward lower wavenumber, as expected for reduction of the [2Fe]^PDT^ cluster, indicative of the H_red_^PDT^ state (Figure S18, Supporting Information).^[13b]^ The nitrile peak of the CNF also shifts to a lower wavenumber (2232.8 cm^−1^, Figure S18, Supporting Information).

**Figure 5:**
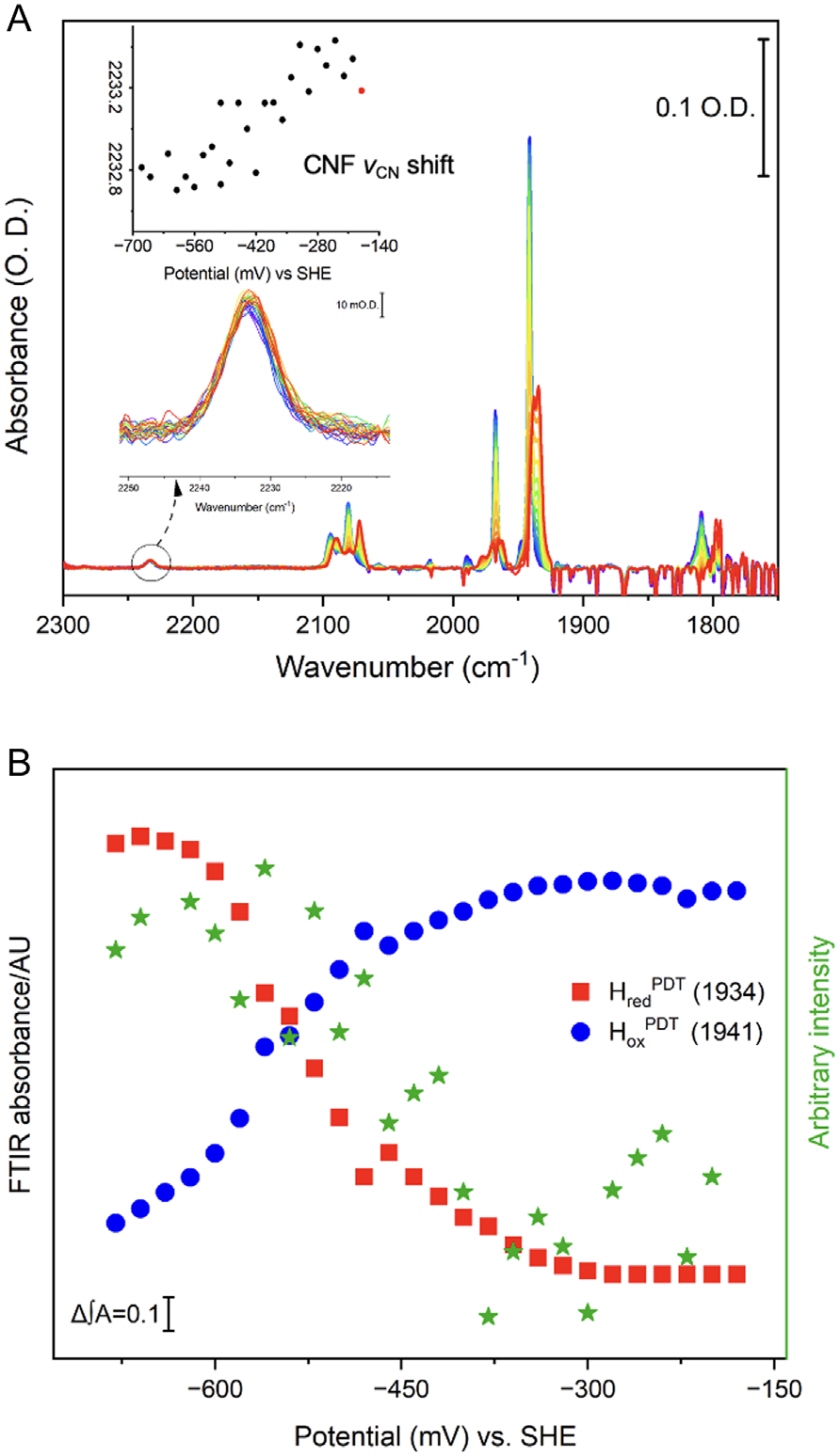
IR spectro‐electrochemistry of F27CNF *Dd*HydAB^PDT^. Spectra were recorded in reflection mode in a reflection‐absorption cell. Conditions: 3.6 mM F27CNF holo‐*Dd*HydAB^PDT^ in 100 mM Tris‐HCl + 150 mM NaCl pH8 buffer containing redox mediators (see Supporting Information, Experimental Section), 1 cm^−1^ resolution, and 1024 co‐averaged scans. The potential was first poised at −160 mV, and then was stepped to −680 mV to reduce the enzyme; an oxidative redox titration was then conducted with the potential stepped back to −180 mV in a series of 20 mV steps. A) Spectra recorded during the oxidative redox titration are rainbow color‐coded from most reduced (red) to most oxidized (blue). Lower inset: zoom‐in of the nitrile stretching spectral region (2200–2300 cm^−1^). Upper inset: nitrile stretching band position versus applied potential; red dot corresponds to the initial poise at −160 mV, and then the black dots correspond to the potential step sequence from −680 back up to −160 mV. B) Left Y‐axis: IR peak intensity versus applied potential for the main CO vibrational bands of H_ox_^PDT^ (1941 cm^−1^, blue), and H_red_^PDT^ (1935 cm^−1^, red). Right Y‐axis: correlation intensity (no units). Autocorrelation intensity of a sample–sample correlation spectrum calculated using the spectra shown in Figure 5. A in the nitrile region (2200–2300 cm^−1^, calculated according to Ozaki and co‐workers).^[^ ^18^ ^]^

Next, we performed an IR spectroelectrochemical redox titration on F27CNF *Dd*HydAB^PDT^ (**Figure** **5**) to compare to WT *Dd*HydAB^PDT^ which has been studied by IR spectro‐electrochemistry.^[13b]^ After recording a spectrum at the start‐potential of –160 mV, the potential was stepped down to −680 mV, and then back up to −180 mV in 20 mV intervals. Spectra recorded during the stepwise oxidative redox titration series (−680 mV to −180 mV) are shown in Figure 5A, colored red through to blue. The H‐cluster^PDT^ CO and CN^−^ stretching bands show the expected conversion of H_red_^PDT^ into H_ox_^PDT^ during the oxidative titration, known to correspond to the removal of an electron from the [4Fe‐4S]_H_ component of the active site H‐cluster. As was observed in the chemical reduction discussed above, the nitrile band of the CNF residue also shifts with applied potential: the initial NC band position when the potential is held at –160 mV is shown as a red dot on the upper inset on Figure 5B and shifts to lower wavenumber when the potential is stepped to ‐680 mV; then there is a gradual shift back to higher wavenumber during the stepwise oxidative redox titration (black dots). Although the magnitude of the overall shift in the nitrile band of CNF between the oxidized and reduced states is less pronounced than in the apo‐protein (only 0.6 cm^−1^) it is clearly reversible on electrochemical reduction/re‐oxidation and matches the shift on chemical reduction, consistent with the CNF sensing redox state in the protein.

The interconversion between H_red_^PDT^ and H_ox_^PDT^ (as observed from the most intense CO band intensities associated with these two states during the oxidative redox titration) is plotted versus the applied potential in Figure 5B (red squares and blue dots respectively; see also Figure S20, Supporting Information) and broadly resembles that reported for WT *Dd*HydAB^PDT^.^[13b]^ The curves are more spread out in potential than would be expected for an ideal Nernstian, 1‐electron process. This is consistent with earlier observations and has been explained by redox changes at the [4Fe–4S]_H_ of the active site influencing the potential of the proximal [4Fe–4S] cluster and vice versa, with both clusters undergoing redox change in this potential range, leading to a complex speciation profile.^[13b]^ Whereas the upper inset in Figure 5B shows the shift in the NC stretching band of CNF with potential, the green stars in Figure 5C show autocorrelation intensity of a sample–sample correlation spectrum calculated using the nitrile region (2200–2300 cm^−1^) of the spectra shown in Figure 5A according to Ozaki and co‐workers^[^ ^18^ ^]^ (see also Supporting Information), and provides confidence that CNF is responding in a meaningful way to the redox changes in F27CNF *Dd*HydAB^PDT^. The interlinked redox behavior of the proximal [4Fe–4S] and the active site [4Fe–4S]_H_ means that we cannot distinguish whether CNF is responding to one in particular of these clusters, and as noted above, the location of CNF is a similar distance from each of these clusters. Redox‐anticooperativity between the electron relay and the H‐cluster, which may influence the electron loading in the electron relay chain of the holo‐protein, may explain the smaller shift in CNF nitrile stretch in the *Dd*HydAB^PDT^ holo‐protein.

Our ability to map the active‐site redox changes via the most intense CO bands from [2Fe]_H_, simultaneously with monitoring the nitrile stretch of CNF, provides confirmation that CNF responds to redox change in the clusters of F27CNF *Dd*HydAB^PDT^.

## 3 Conclusion

In conclusion, we have incorporated the noncanonical amino acid, CNF, into two FeS proteins, and in each case observed a small, reproducible shift (<2 cm^−1^) in the nitrile stretching band of CNF following cluster reduction and re‐oxidation, which is consistent with an electric field effect from proximity to the cluster(s) in different redox states. We have verified that CNF does not alter the native properties of these proteins, using electrochemistry to check that the potential of FdI is unchanged in the CNF variant, and using electrochemistry and IR spectroscopy to confirm that the hydrogenase retains native active‐site states and function. We also obtained high‐resolution crystal structures of CNF‐labeled proteins in both the oxidized and reduced states, demonstrating that site‐specific placement of CNF caused minimal structural perturbation, and giving a precise location for the CNF side chain in each protein. In the multicenter hydrogenase, *Dd*HydAB, the nitrile group of CNF is 12 and 13 Å from the [4Fe–4S] proximal cluster and the [4Fe–4S]_H_ subcluster of the H‐cluster, respectively; the fact that spectroscopic changes are observed at this distance confirm that this functional group has long‐range sensitivity to redox changes. These investigations lay the foundation for employing site‐specifically incorporated CNF to probe the redox state of FeS cluster‐containing proteins by IR spectroscopy. Future studies will attempt to exploit the redox‐induced shift in the NC stretching frequency of CNF to study multicenter enzymes under turnover conditions using operando IR spectroscopy.^[^ ^19^, ^20^ ^]^

## Conflict of Interest

The authors declare no conflict of interest.

## Supporting information

Supplementary Material

## Data Availability

The data that support the findings of this study are available in the supplementary material of this article.
