# Supplementary material for: Cyanophenylalanine as an Infrared Probe for Iron–Sulfur Cluster Redox State in Multicenter Metalloenzymes
Source: Chembiochem. 2025 May 26;26(14):e202500251. doi: 10.1002/cbic.202500251 (PMC12278343; doi:10.1002/cbic.202500251)
Supplement: Supplementary file 1 — Supplementary Material [file CBIC-26-e202500251-s001.pdf]

Supporting Information
©Wiley-VCH 2021
69451 Weinheim, Germany

Cyanophenylalanine as an Infrared Probe for Iron-Sulfur Cluster Redox State in Multi-Centre Metalloenzymes

Zehui Duan,[a‡] Jiaao Wei,[a‡] Stephen B. Carr,[a,b] Miguel Ramirez,[a] Rhiannon M. Evans,[a] Philip A. Ash,[a] Patricia Rodriguez-Macia,[a*] Amit Sachdeva[c*] and Kylie A. Vincent[a*]

SUPPORTING INFORMATION

Table of Contents

Table of Contents *2*

Experimental Procedures *3*

Molecular biology: mutation, expression, and purification 3

Materials and methods 5

Results and Discussion *8*

Figures and Tables *9*

References *25*

Experimental Procedures

Molecular biology: mutation, expression, and purification

FdI

Cloning of plasmids for expression of FdI and its variants in *E. coli*

The FdI gene was cloned downstream, i.e. at the 3'-end, of the gene for GST-tag (glutathione *S*-transferase from *Schistosoma japonicum)* in pGEX6P-1 plasmid (procured from Merck). pGEX6P-1 plasmid was digested using EcoRI (New England Biolabs) and XhoI (New England Biolabs) using manufacturer's recommended protocol. The gene for FdI (obtained as a gene block from FisherScientific) was subsequently inserted into the digested plasmid using Gibson Assembly (New England Biolabs). A short 7 amino acid linker (S-G-G-G-G-G-S) and Strep tag (amino acids W-S-H-P-Q-F-E-K) were also introduced at the 3' end of the synthesised gene with a final stop codon (TAA) to allow purification of full-length, CNF-labelled protein from any protein truncated at the amber stop codon (see below).

pGEX-FdI allows expression of N-terminal GST-tagged-FdI. The protease cleavage site (human rhinovirus 3C and PreScissionTM proteases) between the GST tag and FdI would enable removal of the GST tag from the GST-tagged-FdI protein.

All point mutations to the spinach FdI gene were carried out in the pGEX-FdI plasmid using QuickChange PCR (Agilent). Sites Y3, Y23, Y37, F63, and Y80 were mutated to Y/F/W/A. In addition, sites Y23, Y37, F63, and Y80 were mutated to TAG (amber) stop codon to enable the incorporation of CNF at these positions. Y23, Y37, F63, and Y80 are within 15 Å of the FeS cluster (Figure S1). See Table S1 for primers used in QuckChange PCR. After cloning, the identity of all plasmids was verified using agarose gel electrophoresis and Sanger sequencing (Source Bioscience).

Additionally, for mutants containing the amber stop codon (pGEX-FdI-xxTAG), a C-terminal Step tag was introduced to ensure that the full-length CNF-containing FdI could be separated from the truncated protein. A short 7 amino acid linker (S-G-G-G-G-G-S) was introduced immediately after the FdI gene followed by a Strep tag (amino acids W-S-H-P-Q-F-E-K) and a TAA stop codon. The nucleotides encoding these amino acids were introduced to the pGEX-FdI-xxTAG plasmid using two sequential PCR reactions. First, for each target FdI mutant, the corresponding gene was amplified from the template plasmid using primers FdI_Strep_F and FdI_Strep_R1. After purification, the resulting PCR product was used for a second PCR reaction using primers FdI_Strep_F and FdI_Strep_R2, introducing an XhoI restriction site at the 3' end. The final PCR product was digested with restriction enzymes EcoRI and XhoI and ligated into the pGEX backbone previously digested with the same restriction enzymes. Subsequently, the identity of plasmids was verified using agarose gel electrophoresis and Sanger DNA sequencing (Source Bioscience)

Expression and Purification of non-CNF-containing FdI mutants

For expression of each FdI protein (wild type or point mutants), the corresponding pGEX-FdI plasmid was transformed into BL21 (DE3) *E. coli* cells and cultured in LB medium (Melford) supplemented with 100 µg mL^{-1} ampicillin (referred as LB-Amp after this point) at 37 °C, 220 rpm. This starter culture was then used to inoculate 1 L of fresh LB-Amp media so that OD600 was 0.1. The culture was incubated at 37 °C, 220 rpm until OD600 reached 0.4 to 0.6. At this point protein expression was induced by adding IPTG (1 mM final concentration). After induction, the culture was further incubated at 16 °C with shaking at 220 rpm for 16-18 hrs. Subsequently, cells were harvested by centrifugation at 8000 g for 10 mins, 4°C.

The cell pellet was resuspended in a minimal volume of buffer W (100 mM Tris-HCl, 150 mM NaCl, pH 8.0) supplemented with DNAse I (ca. 30 mg/L, Fisher Scientific) and Lysozyme (from chicken egg white, ca. 120 mg/L, Fisher Scientific). Resuspended cells were lysed by sonication (Fisherbrand™ Q500 Sonicator fitted with standard 0.5-inch probe; 2-second pulse, 5-second pause, total

sonication of 5 minutes) and the cell suspension was centrifuged at 10,000 g, 4ºC for 12 mins to remove the cell debris. The cleared lysate was filtered (0.2 µm filter, Millipore) before being applied to a GST-Trap Glutathione Sepharose 4B column (GE Healthcare), pre-equilibrated with buffer W (see above). Unbound material was washed from the column with passing 5-10 column volumes of buffer W. The column was then conditioned with 10 column volumes of pre-cleavage buffer (50 mM Tris-HCl, 150 mM NaCl, 1 mM EDTA, 1 mM dithiothreitol, pH 7.5) before application of cleavage buffer (1 column volume of pre-cleavage buffer supplemented with 4% (v/v) PreScissionTM protease, 2000 units per µL, Cytiva) and incubation at 4 ºC for 16 hours. The PreScissionTM protease is a fusion protein of human rhinovirus (HRV) 3C protease and GST-tag, allowing for binding of the protease to the GST-trap column. Free (un-tagged) FdI was washed from the column in pre-cleave buffer. The GST-trap column was re-generated by removal of the remaining GST-tag and the bound PreScissionTM protease by washing with 50 mM Tris- HCl, 10 mM reduced glutathione, pH 8.0. The fractions of samples at each stage of purification were analysed for purity by SDS-PAGE (Figure S2).

Each variant was overexpressed following the protocol for spinach ferredoxin explained above. Recombinant FdI and variants were purified by GST affinity chromatography, followed by cleavage of the GST tag, prior to characterisation of the [2Fe-2S] cluster within the variants by UV-Vis spectroscopy and electrochemistry.

For structural and infrared spectroscopic analysis, FdI and its variants were further purified using anion exchange chromatography, and size-exclusion chromatography. After purification using the GST-affinity chromatography, the proteins were loaded onto an anion exchange column (Q Sepharose, GE Healthcare) and eluted using a linear gradient of NaCl from 0 M to 1M (Buffer A: Tris-HCl pH 7.4 with no salt, Buffer B: Tris-HCl pH 7.4 1M NaCl). This resulted in FdI proteins being eluted at ca. 0.55 M NaCl. Further purification by size-exclusion chromatography (Superdex 200 Increase 10/300 GL, GE Healthcare) in 100 mM Tris-HCl, 150 mM NaCl, pH 8.0 resulted in pure FdI protein, as analysed by SDS-PAGE. The molecular weight of FdI is approximately 12 kDa, but the purified protein runs anomalously on SDS-PAGE with a single band at ~24 kDa (Figure S2).

Site-specific incorporation of CNF into FdI

Table S1 provides a summary of primers used for the production of FdI proteins. Position Y37 was selected for incorporation of CNF due to its proximity to the FeS cluster (within 15 Å). In addition, mutating position 37 to W/F/A had minimal influence on the redox and spectroscopic properties of FdI; these variants had wild type-like characteristics.

para-Cyanophenylalanine (CNF) was site-specifically incorporated into FdI in response to the amber stop codon (TAG codon). The pGEX-FdI-37TAG-Step plasmid was prepared as described in the section "Cloning of plasmids for expression of FdI and its variants in E. coli". pULTRA-CNF was employed to provide genetic elements to enable site-specific incorporation of CNF. pULTRA-CNF plasmid contains genes for expression of *Methanococcus janaschii* CNF-specific aminoacyl tRNA synthetase (*Mj*(CNF)RS) and *Mj*$tRNA_{CUA}$.

BL21(DE3) competent cells were co-transformed with pULTRA-CNF and pGEX-FdI-37TAG-Step plasmids. After transformation, the cells were cultured in LB media supplemented with 100 µg mL^{-1} ampicillin and 75 µg mL^{-1} spectinomycin and incubated 37 ℃ with shaking at 220 rpm for 16 h. The culture was then used to inoculate 1 L fresh 2xYT media supplemented with 100 µg mL^{-1} ampicillin and 75 µg mL^{-1} spectinomycin, so that the OD600 was 0.1. This culture was incubated at 37 ºC, 220 rpm until the OD600 reached between 0.4 to 0.6. At this point the protein expression was induced by adding IPTG (1mM final concentration). In addition, the culture was supplemented with 8 mM CNF (Thermo Fisher, final concentration) - a stock of 400mM CNF was prepared in 0.5 M NaOH (in water) and 20ml of this stock was added to 1L culture. The culture was then neutralised by adding 20ml of 0.5M HCL (in water). This culture was further incubated at 16 ℃, 200 rpm for 18-20 hours before the cells were harvested by centrifugation at 8000 g for 10 minutes. Cells were lysed and the lysate cleared as described in the previous section "Expression and Purification of non-CNF-containing FdI mutants". The full-length protein was purified by strep-tactin affinity chromatography: The supernatant was loaded onto a column equilibrated by buffer W (see previous section "Expression and Purification of non-CNF-containing FdI mutants"), followed by a washing with buffer W before the desired protein was eluted in 100 mM Tris-HCl pH 8.0, 150mM NaCl, 2.5 mM desthiobiotin. The eluted fractions were analysed by SDS-PAGE.

DdHydAB

Molecular biology details about mutant F27CNF apo-*Dd*HydAB

pACYCDuet-1 apo-*Dd*HydAB plasmid was mutated to introduce amber stop codon (TAG) at position 27. This was achieved using QuickChange PCR (Agilent), resulting in plasmid plasmid, pACYCDuet-apoHydAB-27TAG. Subsequently, the DNA sequence for Step tag was inserted downstream of the apo-*Dd*HydAB gene.

Expression of wild-type and F27CNF apo-*Dd*HydAB

BL21(DE3)*ΔiscR* cells were transformed with pULTRA-CNF and pACYCDuet-apoHydAB-27TAG plasmids and the cells were transferred to LB media supplemented with 50 μg mL^{-1} kanamycin, 35 μg mL^{-1} chloramphenicol, and 50 μg mL^{-1} spectinomycin. Note that BL21(DE3)*ΔiscR* cells have kanamycin resistance marker, whereas pULTRA-CNF and pACYCDuet-apoHydAB-27TAG plasmids have spectinomycin and chloramphenicol resistance markers. The culture was incubated overnight (12-16 h) at 37ºC, 220 rpm. Next day, this starter culture was used to inoculate 1 L of LB-phosphate media (20 g L^{-1} LB, 10 g L^{-1} K_2HPO_4, 10 g L^{-1} Na_2HPO_4), supplemented with 50 μg mL^{-1} kanamycin, 35 μg mL^{-1} chloramphenicol, and 50 μg mL^{-1} spectinomycin. In addition, the media was supplemented with 2 mM ammonium ferric citrate and 2 mM cysteine, as the sources of Fe and S for FeS clusters in the protein, and 0.5% w/v glucose. The culture was grown at 37 ºC and 200 rpm till the OD600 reached around 0.7. Subsequently, the culture was cooled down in a room-temperature water bath for 10 min. A 200 mM stock of CNF was prepared in 0.5 M NaOH (in water) and added to the main culture so that the final concentration of CNF in the culture is 3 mM. The culture was neutralised to pH~7 by adding the same amount of 0.5 M HCl (in water). This culture was then purged with argon for 1 hour to create anaerobic conditions. Subsequently, the culture was supplemented with 1mM IPTG (final concentration) to induce protein expression. An additional 3 mM L-cysteine was added, and the culture was further incubated at room temperature with stirring for 16-20 hrs. Cells were harvested anaerobically by centrifugation (13000 g, 35 min, 4℃), and the cell pellet was either used immediately for protein purification or stored at -80℃ until purification.

For protein purification, inside the glovebox, the cell pellet was resuspended in Tris buffer (pH 8, 100 mM Tris and 150 M NaCl) supplemented with protease inhibitor cocktail (Sigma). The resuspended pellet was sonicated (5 x 3 min with 30% amplitude and 50% duty, 1 second one 1 second off, with 5 min waiting between each round of 3 min sonication for cell lysis) and centrifuged (320000 g, 1hr, 4℃). The supernatant containing the apo-protein was then purified via a strep-tag affinity column and analysed using SDS-PAGE (Figure S11). 10-11 mg and 3-4 mg of apo-*Dd*HydAB and F27CNF apo-*Dd*HydAB were obtained from 1 L culture, respectively.

Materials and methods

Ultraviolet/Visible (UV/Vis) Characterisation

UV/vis spectroscopy was applied to measure the correct assembly of iron-sulfur clusters in (CNF) FdI and apo-*Dd*HydAB. The UV/vis spectra were recorded by a Cary 60 spectrophotometer (Agilent Technologies) with a cuvette (path length 1 cm, cell measure volume 100 uL, Fisher Scientific), against a baseline of the corresponding buffer. The scan region is 200 to 800 nm. The concentration of *Dd*HydAB was calculated based on the average of absorbance at λ = 280 nm (ε_{280} = 52.5 mM^{-1} cm^{-1}) and λ = 400 nm (ε_{400} = 143 mM^{-1} cm^{-1}), which indicates the aromatic residues within the protein backbone and the ligand to metal charge transfer (LMCT) of the [4Fe-4S] cluster.

Transmission FTIR spectroscopy

IR spectra were recorded on a Bruker Vertex 80 FTIR spectrometer controlled by OPUS software, equipped with a mercury cadmium telluride (MCT) detector cooled with liquid N2 housed inside a dry, anaerobic glovebox (Glove Box Technology Ltd. UK, O_2 less than 2

ppm). The IR transmission cell was assembly in the same anaerobic glovebox. The protein sample (3 µL, 1 to 2 mM) was placed in between two CaF_2 windows (31.8 mm x 1.5 mm, Crystran) separated by a 25 µm Teflon spacer (PIKE Technologies) coated in vacuum grease and then sealed on a commercial IR transmission cell (PIKE). All the measurements for F27CNF *Dd*HydAB were recorded at 1 cm^{-1}, for FdI protein and WT *Dd*HydAB, at 2 cm^{-1}. And 1024 scans were co-averaged.

Protein Film Electrochemistry (PFE)

PFE was carried out on FdI variants, WT *Dd*HydAB^{ADT} and variants. The catalytic features of F27CNF *Dd*HydAB^{ADT} and of WT *Dd*HydAB^{ADT} (used as a control) are identical, confirming that the introduction of CNF does not impact the catalytic activity of the enzyme. The main electrochemical cell compartment held around 10 mL buffer mix, composed of 15 mM of each MES (2-[N′-morpholino]-ethane sulfonic acid), HEPES (N′-[2-hydroxyethyl]piperazine-N′-2-ethane sulfonic acid), TAPS (N′-tris[hydroxymethyl]methyl-3-amino propane sulfonic acid), CHES (2-[N′-cyclohexylamino]ethane sulfonic acid) (all from Melford) and sodium acetate (Sigma), with 0.1 M NaCl (Fisher) as supporting electrolyte, prepared using purified water (Millipore: resistivity 18.2 MΩ cm) and titrated with HCl to pH 6.0 at 25 °C. Buffer was flushed with N_2 overnight to remove O_2 before being taken into the glove box. The protein solution (4 µL, 6 µM, in buffer 10 mM MES pH 5.8) was directly placed on the surface of a pyrolytic graphite 'edge' (PGE) working electrode (area = 0.03 cm^2, polished by P2500 sandpaper) and left for three minutes for direct enzyme absorption to form the protein film on the PGE surface before unabsorbed enzyme was rinsed away using purified water. Pt wire was used as counter electrode. Saturated Calomel Electrode (SCE) was used as a reference and was held in an isolated glass side-arm containing 0.10 M NaCl and connected to the main cell compartment by a Luggin capillary. The SCE was calibrated with ferrocene-methanol as +241 mV versus the standard hydrogen electrode (SHE) at 25 °C, and potentials are corrected back to potential (unit mV) vs SHE in all figures. The electrical current was monitored versus the applied potential using an Autolab PGSTAT128N potentiostat using NOVA software version 1.10. Experiments were conducted in a three-electrode electrochemical cell housed inside an anaerobic glovebox (Glove Box Technology Ltd. UK, O_2 < 2 ppm). The main cell compartment was surrounded by a water jacket for temperature control. Cyclic voltammetry experiments were performed at 25 °C. Electrode rotation was controlled by a Metrohm Autolab IME663 rotator, and the electrode was rotated at 2000 rpm in all experiments shown here in order to achieve effective mass transport to/from the immobilised enzyme film. All experiments on hydrogenase were conducted with 1000 scc/min H_2 (BOC) flushing through the headspace of the electrochemical cell, using gas mass flow controllers (Smart-Trak2, Sierra Instruments).

Solution activity assay

Solution activity assays were performed on wild-type *Dd*HydAB^{ADT} and F27CNF *Dd*HydAB^{ADT} using a redox active dye chosen to have a midpoint potential that matches *Dd*HydAB^{ADT}: benzyl viologen (BV, E^o= - 0.350 V, vs SHE). The reduction of 1 mM BV by *Dd*HydAB^{ADT} was monitored at 600 nm ((ε_{600} = 7.0 mM^{-1} cm^{-1}) by UV/vis spectroscopy, in H_2-saturated 50 mM pH 8 Tris buffer, at 25℃. 3 repeated measurement were performed on each protein sample to ensure reproducibility and to calculate error bars.

IR spectroelectrochemistry

IR spectroelectrochemical experiments were performed using a Bruker VERTEX 70 FT-IR spectrometer equipped with a liquid nitrogen cooled Bruker mercury cadmium telluride (MCT) detector with a home-built electrochemical reflection IR cell, coupled to a Hyperion 3000 IR microscope (Bruker). Sample (20 µL) of 1.5 mM F27CNF apo-*Dd*HydAB or 3.6 mM F27CNF *Dd*HydAB^{PDT}, containing 0.5 mM of the redox mediators anthraquinone-2-sulfonate (E_{m7} = −277 mV), benzyl viologen (E_m = −358 mV), methyl viologen (E_m = −449 mV), 1,2-Bis(2-aminophenoxy) ethane-N,N,N',N'-tetraacetic acid, europium(III) chloride, and 1 mM reduced myoglobin as O_2 scavenger, was loaded on the glassy carbon working electrode of the microspectroscopic-electrochemical cell. A thin CaF_2 window (0.5-1 mm, Crystan) was sealed onto the cell surface with a 25 µm Teflon spacer in between. A carbon counter electrode and a miniature Ag/AgCl (sat. KCl) reference electrode completed the three-electrode system. The reference electrode was calibrated before and after each measurement using ferrocene-methanol (E_m = +436 mV) versus the standard hydrogen electrode (SHE) at 25 °C. Assembly of the reflection IR microspectroscopic-electrochemical cell and mediator solution preparation, were carried out in a N_2-filled glovebox (Glove Box

Technology Ltd. UK, O_2 less than 2 ppm). Spectra were recorded as an average of 1024 scans working at 1 cm^{-1} resolution, with applied potential by an Autolab PGSTAT128N controlled by Nova software version 1.10.

Infrared spectroscopic data workup

For all the IR spectra of *Dd*HydAB proteins and the transmission IR spectra of labelled FdI protein, baseline subtraction was conducted for each spectrum using an interpolated spline function in Kazan by carefully putting as few anchor points as possible, to limit the influence of the anchor points on the peak shape. The baseline subtracted spectra were then transferred to the spectral analysis program Orange Quasar,[1] to analyse the precise position of the vibrational stretching band of nitrile, as well as the band area of the state marking CO stretching bands of F27CNF *Dd*HydAB^{PDT} at 1941 cm^{-1} and 1935 cm^{-1} and give the peak area and plot against applied potentials. The obtained processed spectra and peak information were consequently transferred to Origin to generate plots. For a better fitting for the nitrile stretching band of CNF, the Voigt peak fitting profile model in Orange Quasar, as a combination method of Gaussian and Lorentzian models, was chosen. Voigt is suitable for liquids, because molecular interactions prevent extremely rapid motion but are not locked in place, therefore leading to a curve shape with the features of both Gaussian and Lorentzian character.[2] Voigt fitting was performed with limits centre (2230 to 2235), flexible amplitude and limits *sigma* (1, limits from 0 to 3), *gamma* was default constrained to *sigma*, as the full width at half maximum is approximately 3.6013 *sigma*. However, Voigt fitting model shows very little improvement for the state marking v_{CO} bands compared with Gaussian model. In this case, Gaussian model was used with limits centre, flexible amplitude and width. For the reflection IR of CNF FdI, due to the low peak intensity, the vibrational stretching band of nitrile (*p*CNF) could only be identified after the second derivative of the original spectra. The second derivative of the IR spectra shows a small peak at around 2236 cm^{-1}, and this peak is fitted by the second derivative of Gaussian with the same restrictions applied above.

Crystallisation

Purified Fd1 was concentrated to 35-40 mg mL^{-1}, as assessed by Bradford assay, using an Amicon centrifugal filtration unit (Merck Millipore, Germany) with a molecular weight cutoff of 10 kDa. Crystallisation, using the sitting drop vapour diffusion technique, was performed by mixing 100 nL of protein solution with an equal volume of crystallisation solution (100 mM Na, K Phosphate pH 7-8, 2.6-3.6 M Ammonium Sulphate) using a mosquito crystallisation robot (SPT labtech, UK) followed by incubation at 277 K. Crystals were transferred to cryoprotecting solution (100 mM Na, K Phosphate pH 7.5, 3.6 M Ammonium Sulphate, 5% v/v glycerol) for ~15 seconds then flash cooled by plunging into liquid nitrogen. Crystals of oxidised ferredoxin were produced by addition of 10 mM potassium ferricyanide to the cryoprotectant solution, whereas crystals of reduced ferredoxin were obtained by transferring them to an anaerobic chamber overnight (nitrogen atmosphere O_2 < 0.3ppm) followed by soaking in oxygen–free cryoprotectant also containing 10 mM dithionite for several minutes prior to flash cooling.

All stages of the crystallisation process for *Dd*HydAB were performed in a nitrogen filled glovebox (nitrogen atmosphere O_2 <0.3ppm) under strictly anaerobic conditions. Purified *Dd*HydAB was exchanged into 10 mM Tris.HCl pH 7.6 buffer and concentrated to 15 mg mL^{-1} (270 µM). Crystals were obtained using sitting drop vapour diffusion by mixing 2 µL of protein solution with an equal volume of crystallisation buffer (0.9 M $LiSO_4$, 0.1 CH_3COONa, 24-30% w/v PEG 6000) and allowed to equilibrate against a 500 µL reservoir at 23 °C (ambient temperature of glove box). Crystals were cryoprotected by transferring into crystallisation buffer containing 50% w/v PEG 6000 supplemented with 10 mM sodium dithionite or 10 mM potassium ferricyanide to produce reduced or oxidised protein respectively.

X-ray diffraction data were collected at beamlines I03 and I24 (Diamond Light Source, UK) with details summarised in Table S3. Diffraction data were reduced using either Dials[3] or Autoproc.[4] Phase estimates were generated by Molecular Replacement using Phaser[5] with native spinach ferredoxin-I (pdb id 1A70) or *Dd*HydAB (pdb id 6SG2) as a search model. Iterative rounds of rebuilding in Coot[6] and refinement with either Refmac[7] or Phenix[8] produced the final models reported herein. The diffraction data for oxidised FdI Y37F displays a significant level of pseudo-merohedral twinning, which was accounted for during structure refinement. All resultant models were validated using the Molprobity server[9] .

Differential Scanning Fluorimetry

Protein solution at 1 mg mL^{-1} was loaded into capillaries and melting temperature experiments were performed using a Prometheus nanoDSF (Nanotemper, Germany) using a temperature ramp of 1 °C per minute. Protein unfolding was monitored by measuring tryptophan fluorescence at 330 and 350 nm and melting temperature was assessed from the second derivative of the melting curve.

Results and Discussion

Electrochemical and UV-visible spectroscopic characterisation of non-CNF-labelled FdI proteins

The nature of the [2Fe-2S] of FdI proteins was investigated first by UV-vis spectroscopy and electrochemistry. The characteristic peaks in the UV-vis spectrum can be seen at 420-460 nm with a shoulder at 320 nm for both the oxidised native FdI (Figure S4) and oxidised variant Y37F (Figure S5). These absorption peaks were lost when the proteins were reduced with a stoichiometric amount of sodium dithionite.

The redox properties of the FeS cluster of FdI proteins were assessed by electrochemical analysis. Cyclic voltammetry on FdI was performed in 150 mM Tris-HCl pH 8 buffer containing 100 mM NaCl, using a glassy carbon working electrode, in an electrochemical cell equipped with a miniature Ag/AgCl reference electrode and carbon counter electrode. Potentials are converted to V versus the standard hydrogen electrode (SHE) using the correction *E*(SHE) = *E*(Ag/AgCl) + 0.20 V. Well-defined diffusion-controlled reduction and oxidation peaks were observed with a peak-to-peak separation of ca. 100 mV and a midpoint potential of –0.405 V (Figure S3).

Producing of F27CNF *Dd*HydAB^{ADT} and F27CNF *Dd*HydAB^{PDT}

Two [FeFe] active site mimics, $[Fe_2(adt)(CO)_4(CN)_2]^{2-}$ and $[Fe_2(pdt)(CO)_4(CN)_2]^{2-}$, were incorporated into the F27CNF apo-*Dd*HydAB protein scaffold. F27CNF *Dd*HydAB^{ADT} contains the native-like $[2Fe]_H$ cluster and can be used to study the catalytic mechanism. To simplify the redox states, the $[2Fe]_H$ can be made catalytically inactive by substituting the amine group (ADT) with methylene group (PDT) (Figure S14). This modification does not affect the electron transfer steps at the electron relay but prevents the $[2Fe]_H$ subcluster from being reduced as it cannot become protonated. Desirably, it simplifies the system as F27CNF *Dd*HydAB^{PDT} can only cycle cleanly between two redox states of H_{ox}^{PDT} (oxidised state) and H_{red}^{PDT} (reduced state).

Crystallography of FdI and *Dd*HydAB

Crystals of FdI appeared overnight with native and CNF containing variants both producing large rhombohedra. Although the crystallisation conditions were similar to those previously published[10] the crystal symmetry and unit cell parameters varied significantly between variants and redox state, however, this had little impact on the diffraction quality of the crystals (Table S3) with all samples diffracting to near atomic resolution. Large plate-like crystals of *Dd*HydAB, which again diffracted x-rays to very high (~1 Å) resolution, appeared after 2-3 days. Given the high resolution of the x-ray data the resulting electron density maps were of exceptional quality, clearly displaying each of the introduced mutations (Figure S16) and allowing their position to be determined with high precision.

Superposition of the native and CNF containing variant structures for each protein (Figure S2, 3) shows that changing the identity of the amino acid at either position 27 or 37 has no effect on the structure of the protein either at a global or local level, with each CNF side chain adopting the same conformation and F/Y in the native enzyme. Furthermore, changing the oxidation state of the FeS clusters also has no effect on the position of the side chains relative to the metallo-cofactors. Analysis of the temperature factors of amino acids surrounding the mutated residues shows the mutations do not alter the flexibility of the protein in these regions either.

Although diffraction is observed to ~1 Å for each crystal system, this is insufficient resolution to observe changes in the Fe-S bond lengths within the FeS clusters. Previous ultra-high resolution structures of the bond lengths in High-Potential Iron-sulfur Protein (HiPIP)[11] show that Fe-S bond lengths of the 4Fe4S cluster change slightly upon oxidation/reduction (0.005-0.01 Å). This suggests similar small changes likely occur in the FeS clusters of FdI and *Dd*HydAB, but are not observed since the changes are less than the precision of the atomic coordinates (0.02 Å)

Spectro-electrochemistry on F27CNF *Dd*HydAB

Figure S20 demonstrates the full reversibility upon F27CNF *Dd*HydAB^{PDT} electrochemical reduction and re-oxidation. Spectra align well with the spectro-electrochemistry of wild-type *Dd*HydAB^{PDT} published previously.[12] The crossing point of reduced and oxidised species is shifted according to the potential titration direction (hysteresis), but this shift aligns with the [4Fe-4S]$_H$ redox behaviour under the influence of intercluster redox coupling with the other two [4Fe-4S] clusters in the electron relay.[12] Some slight drift in reference electrode potential and pH is likely during these long redox titrations and may also contribute to the hysteresis.

The secondary y-axis in Fig 5C of the main text is a measure of the autocorrelation intensity (unitless) calculated as the diagonal of the covariance of the experimental spectra in the nitrile stretching region (2200-2300 cm-1) expressed as a m by n matrix Y, where each column, m, represents the dynamic spectral response at a given wavenumber across n applied potentials. The covariance expressed in this form is termed the synchronous sample-sample correlation spectrum (Φss). See reference.[13]

Supplementary Figures and Tables

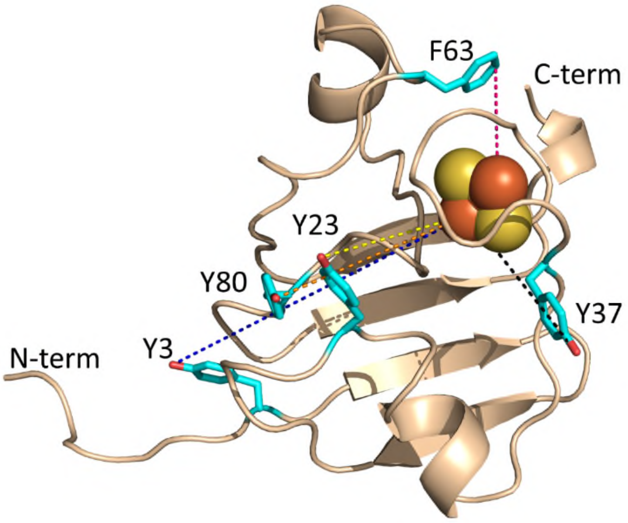

Figure S1. The structure of native spinach FdI (this work) is shown as a cartoon representation with the residues of interest selected for mutation shown as sticks (Y3, Y23, Y37, F63, and Y80). The [2Fe-2S] cluster is shown as spheres. The distances of each residue of interest to the nearest Fe atom of the [2Fe-2S] cluster are shown as dashed lines as follows Y3 (blue = 22.3 Å), Y23 (yellow = 9.9 Å), Y37 (black = 10.7 Å), F63 (pink = 8.7 Å) and Y80 (orange = 12.7 Å). Prepared using PyMOL 4.50.

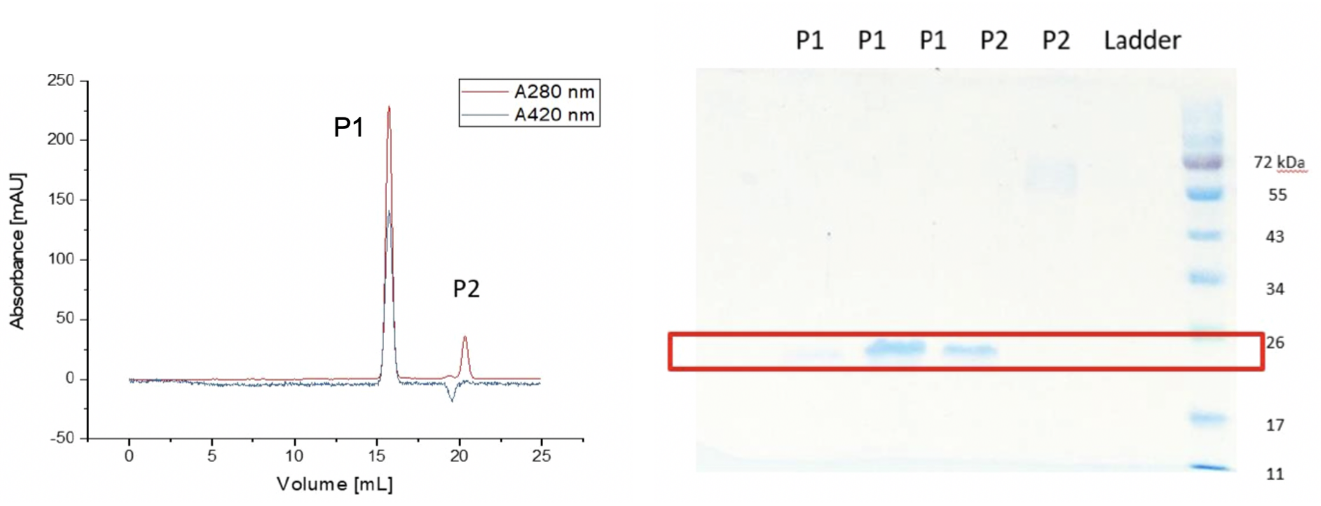

Figure S2. Size-exclusion purification result of wild-type FdI protein. Following GST-trap and Q-sepharose purification, wild-type FdI protein (Top) resulted in two peaks eluting from the column (P1 and P2) with only P1 having an absorbance at 420 nm (indicating the presence of the FeS cluster). The corresponding SDS-PAGE electrophoresis of the protein fractions was collected during size-exclusion chromatography (bottom). The molecular weight of FdI is approximately 12 kDa, but the purified protein runs anomalously during SDS-PAGE electrophoresis with a single band at ~24 kDa.

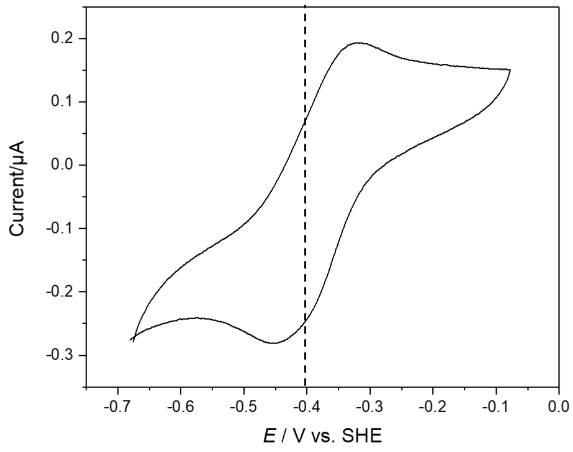

Figure S3. Cyclic voltammogram for a solution of wild-type FdI at a glassy carbon working electrode in 100 mM Tris-HCl pH 8, 150 mM NaCl at 25 ^{o}C, recorded at 10 mV s^{-1}. The dashed line indicates the midpoint potential (-0.405 V).

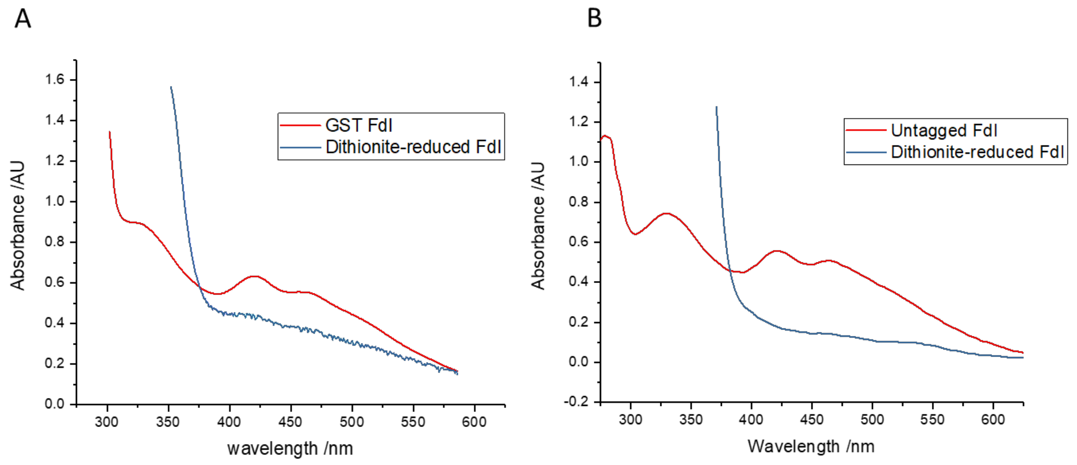

Figure S4. UV-visible spectra of oxidised and dithionite-reduced FdI. In 100 mM Tris-HCl buffer, pH 8: (A) with the GST-tag in place, and (B) after removal of the GST-tag.

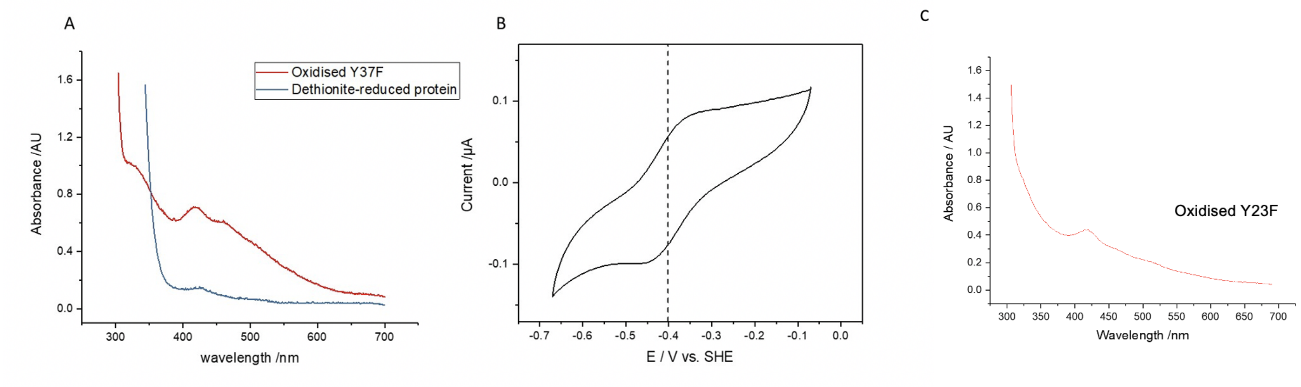

Figure S5. Selected UV-visible spectra and cyclic voltammogram. (A) UV-visible spectrum of oxidised (red) and reduced (blue) Y37F FdI and (B) cyclic voltammogram for Y37F FdI, showing that these are indistinguishable from the native-sequence FdI. C) UV-visible spectrum (oxidised) Y23F variant, showing abnormal cluster absorption features.

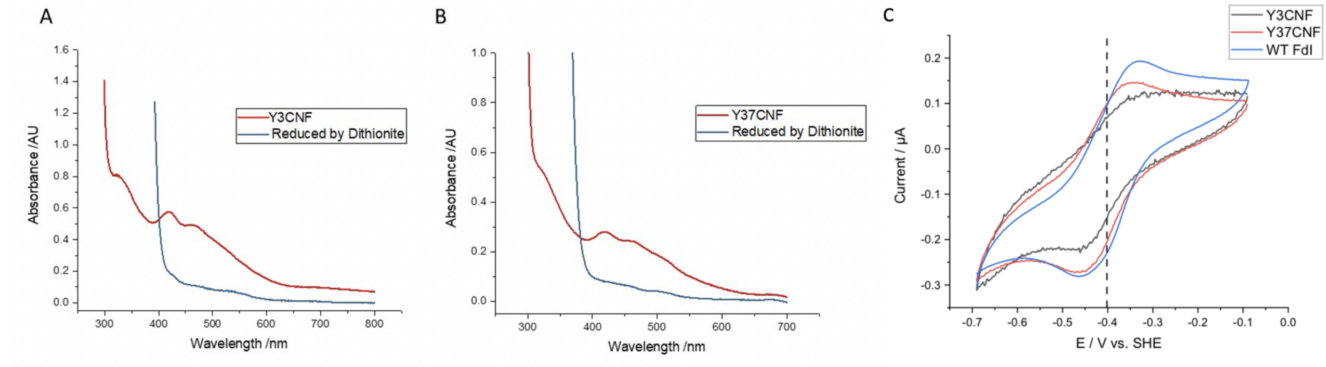

Figure S6. Representative UV-visible spectra and cyclic voltammogram for FdI Y3CNF and Y37CNF variants. A: Y3CNF FdI purified by Strep-tactin affinity chromatography. B. Y37CNF FdI purified by Strep-tactin affinity chromatography. The red traces correspond to the oxidised ferredoxin in 100 mM Tris-HCl pH buffer; the blue traces represent the dithionite-reduced protein in 100 mM Tris-HCl pH 8 buffer. Cyclic voltammograms of the wild-type FdI (blue line), Y3CNF variant (black line) and Y37CNF variant (red line) measured in solution at a glassy carbon working electrode in 100 mM Tris-HCl 150 mM NaCl buffer pH 8, 25 ^{o}C.

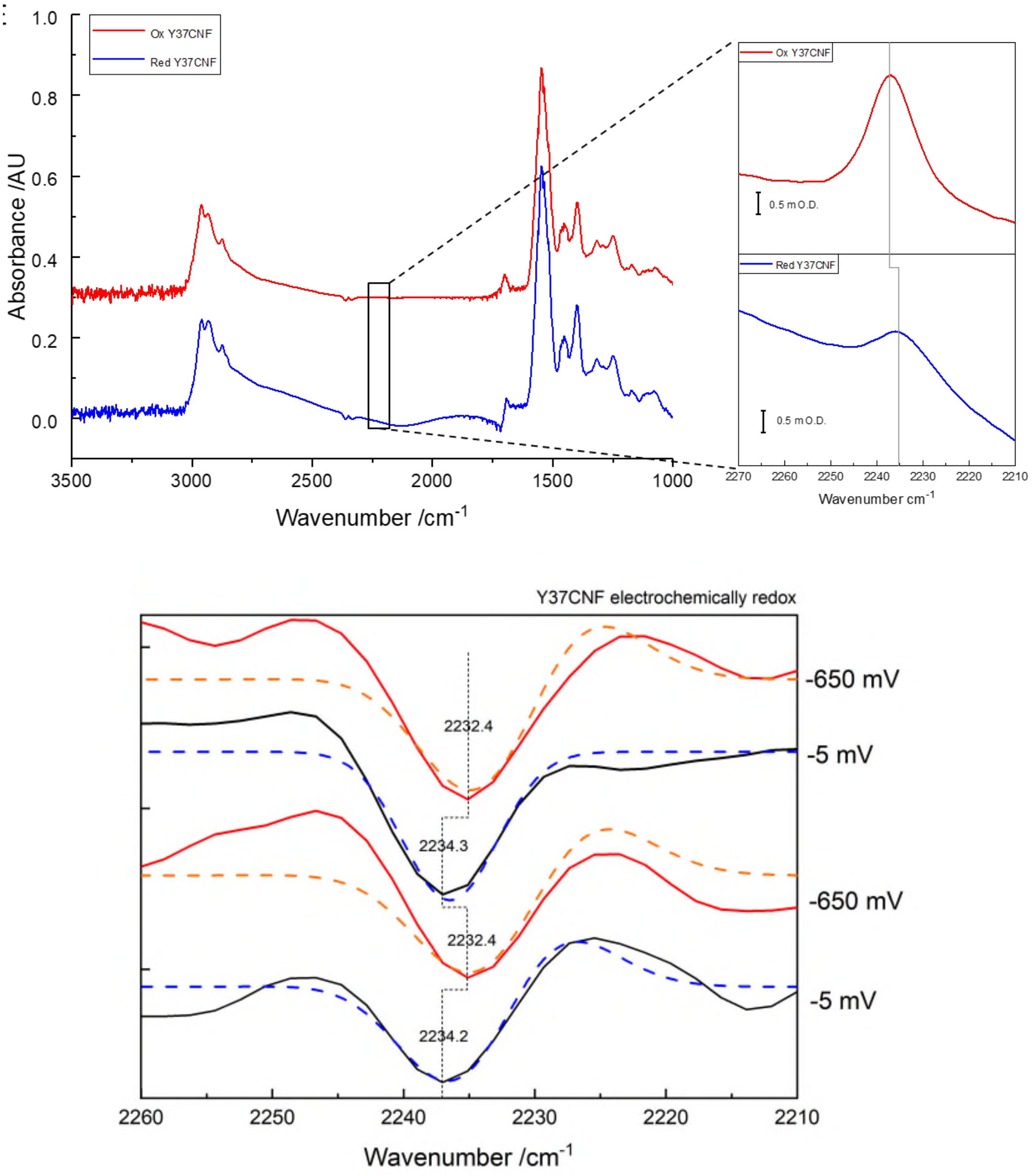

Figure S7. Upper panel: IR spectra in reflection-absorption mode of Y37CNF FdI under electrochemical control. The inset enlarges the nitrile stretching region, revealing a small shift in the position of the nitrile stretching band. The lower panel (solid lines) show second derivative data for spectra in the nitrile region over 2 cycles of reduction / re-oxidation; dashed lines show fitting of a second derivative Gaussian function.

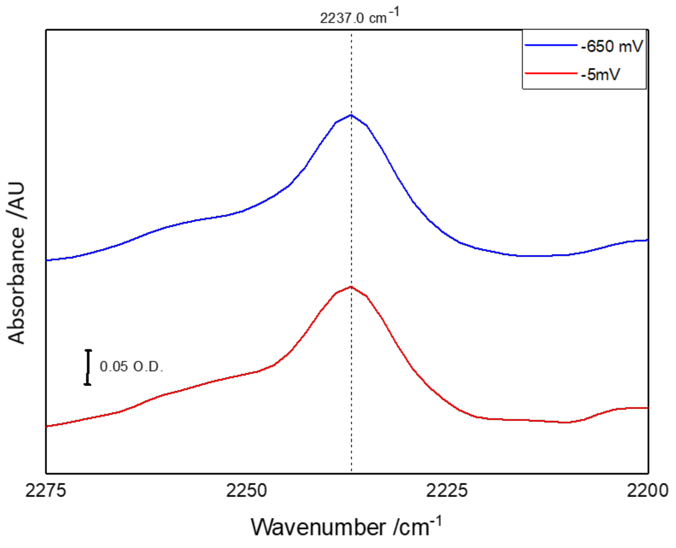

Figure S8. Control experiments showing baseline-subtracted IR spectra for 100 mM *para*-cyanophenylalanine (CNF). In 100 mM Tris-HCl buffer, pH 8, containing 150 mM NaCl. These were measured in the reflection-absorption mode in a spectroelectrochemical cell which has been reported previously.[14] Red spectrum: -5 mV; blue spectrum: -650 mV. Conditions: 25 °C, 2 cm^{-1} resolution, 1024 co-averaged scans.

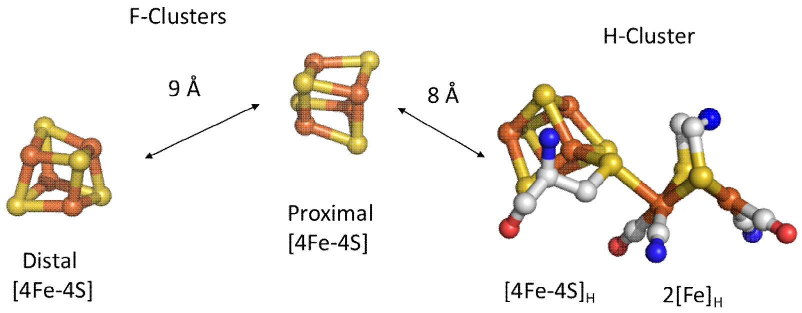

Figure S9. Arrangement of clusters in *Dd*HydAB^{ADT}. Yellow spheres represent sulfur atoms, brown spheres represent iron atoms, grey spheres represent carbon atoms, red spheres represent oxygen atoms, blue spheres represent nitrogen atoms. (PDB code: 1HFE.)

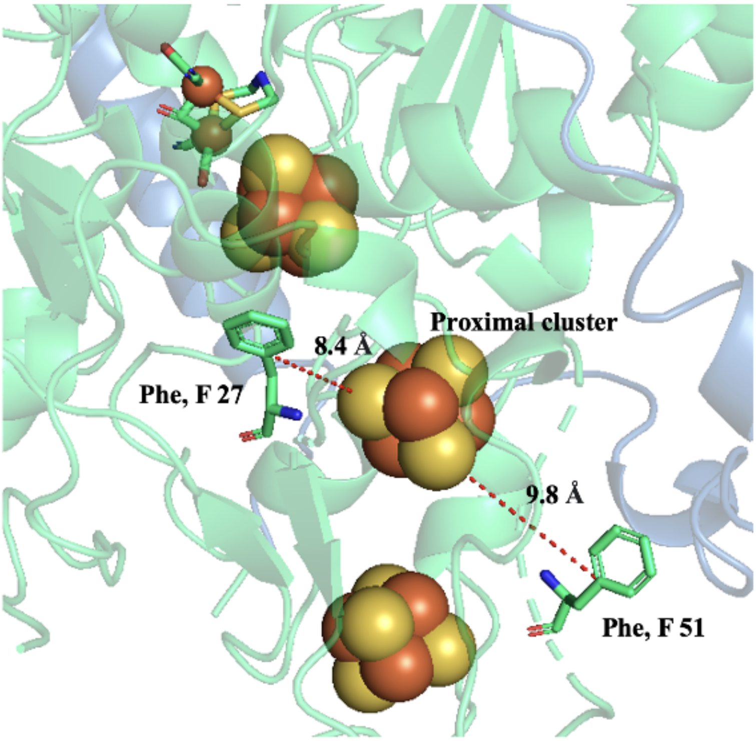

Figure S10. Structure of *Dd*HydAB prepared from PDB code 1HFE. The green cartoon represents the large subunit HydA and the blue cartoon represents the small subunit HydB. [4Fe-4S] clusters and the [2Fe]$_H$ subcluster are represented by spheres, with the yellow ones being the S atoms and the orange ones being the Fe atoms. Phe27 and Phe 51 were considered as possible sites for mutagenesis to CNF.

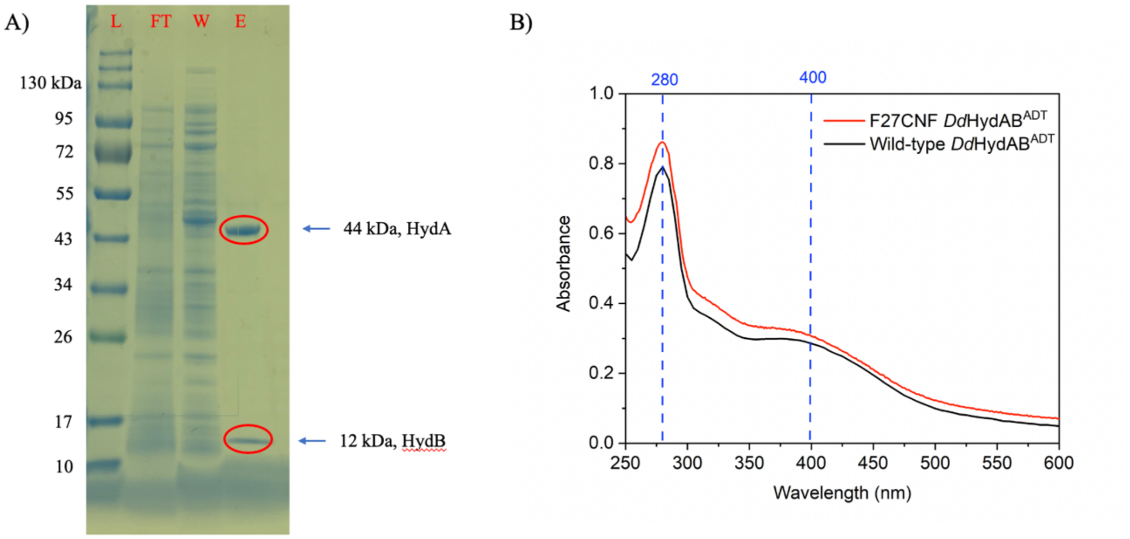

Figure S11. (A) The SDS-PAGE of the F27CNF apo-*Dd*HydAB hydrogenase after strep-tag affinity column. The lanes are (from left to the right) L-lysate, FT-flowthrough, W-wash, E-elution. The elution lane only contains two bands corresponding to the two subunits of apo-*Dd*HydAB, HydA (44 kDa) and HydB (12 kDa). (B) UV/vis spectrum of purified proteins, wild-type *Dd*HydAB^{ADT} and F27CNF *Dd*HydAB^{ADT}, indicate correct assembly of the iron-sulfur clusters.

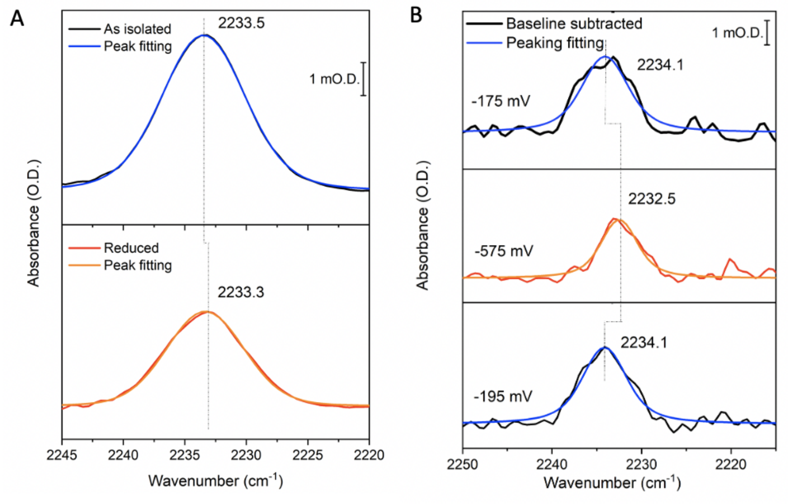

Figure S12. (A) IR spectra of F27CNF apo-*Dd*HydAB in the as-isolated state, before and after addition of 10 mM sodium dithionite, with Voight-profile peak fitting for the CN vibrational band (around 2234 cm^{-1}). (B) IR Spectroelectrochemistry of F27CNF apo-*Dd*HydAB. Spectra were recorded in reflection mode. Conditions: 1.5 mM F27CNF apo-*Dd*HydAB in 100 mM Tris-HCl + 150 mM NaCl pH8 buffer containing redox mediators (see experimental section), with Voight-profile peak fitting, 1cm^{-1} resolution, 1024 co-averaged scans.

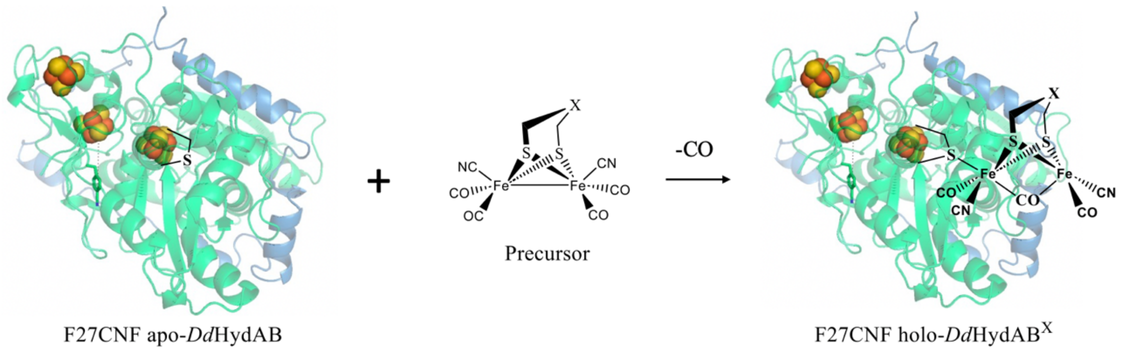

Figure S13. Schematic representation of artificial maturation of F27CNF apo-*Dd*HydAB, X = NH (for ADT), CH_2 (for PDT).

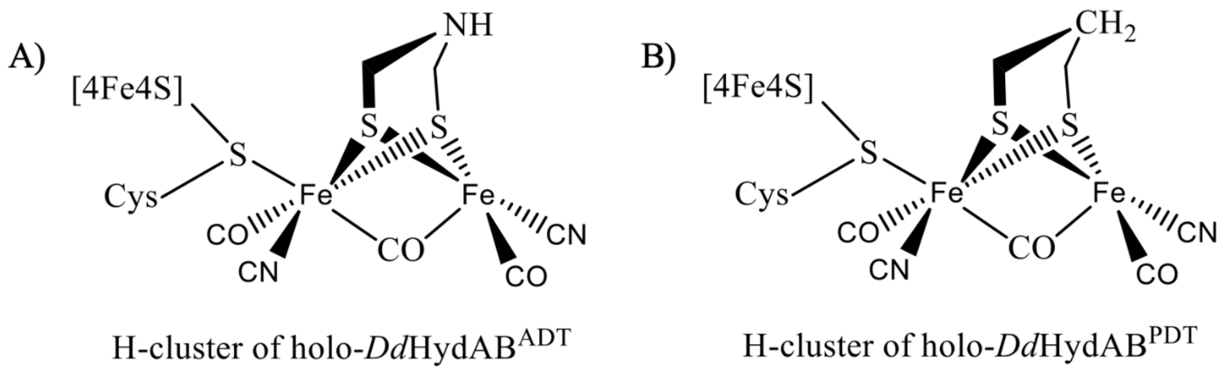

Figure S14. Structure of the H-cluster in the wild-type *Dd*HydAB^{ADT} (A) and in the *Dd*HydAB^{PDT} variant (B).

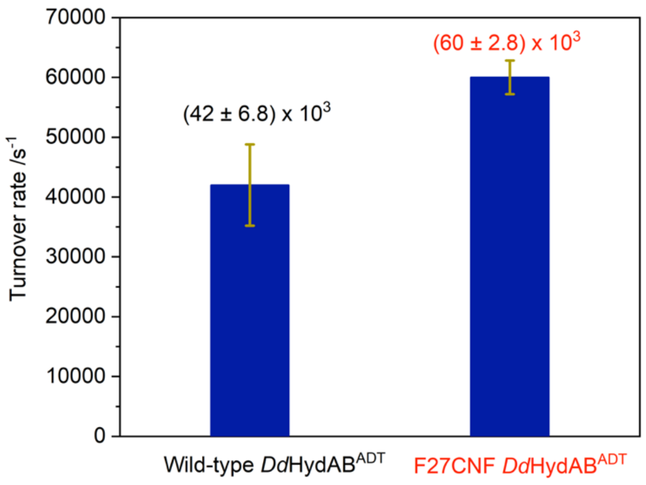

Figure S15. Comparison of the H_2 oxidation activity of wild-type *Dd*HydAB^{ADT} and F27CNF *Dd*HydAB^{ADT}. *Dd*HydAB^{ADT}: (42 ± 6.8) x 10^3 s^{-1}; F27CNF *Dd*HydAB^{ADT}: (60 ± 2.8) x 10^3 s^{-1}. Turnover rates and the error bars were calculated from 3 repetitions of each activity measurement, recorded by UV/Vis. with colour change of redox dye measured at 600 nm, 25 ℃.

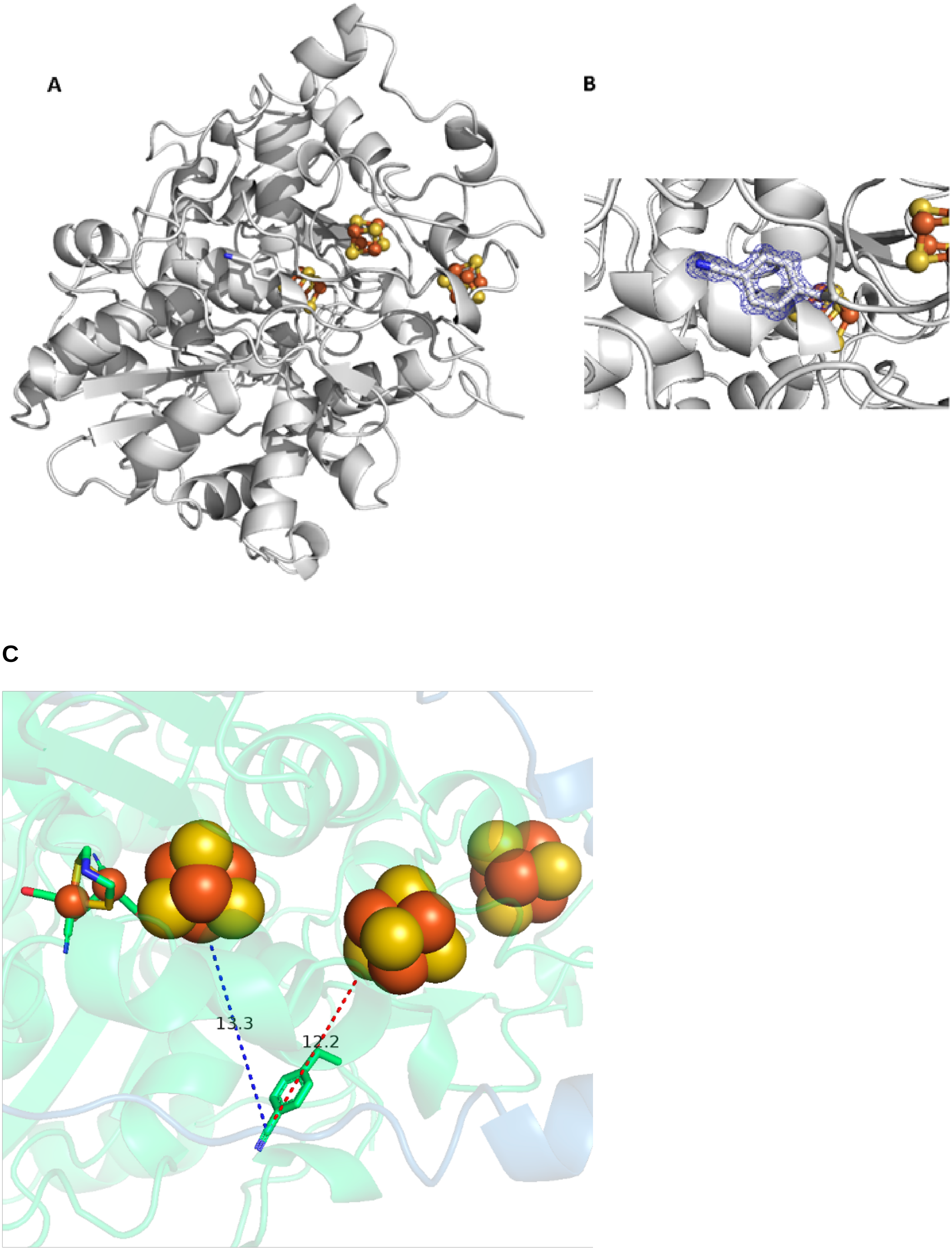

Figure S16. (A) Structure of F27CNF apo-*Dd*HydAB; (B) 2Fo-Fc electron density for apo-*Dd*HydAB (blue mesh) at position 27 demonstrating incorporation of the CNF label. (C) The actual distances measured from the crystal structure of F27CNF *Dd*HydAB^{PDT} between the FeS clusters and the NC group of CNF; the green cartoon represents the large subunit HydA and the blue cartoon represents the small subunit HydB. [4Fe-4S] clusters and the $[2Fe]_H$ subcluster are represented by spheres, with the yellow ones being the S atoms and the orange ones being the Fe atoms.

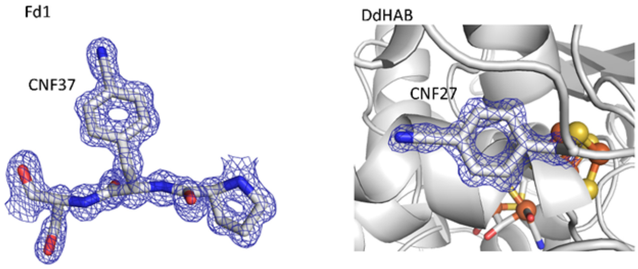

Figure S17. Nitrile groups are clearly visible in 2Fo-Fc electron density maps calculated for Fd1 Y37CNF and F27CNF *Dd*HydAB.

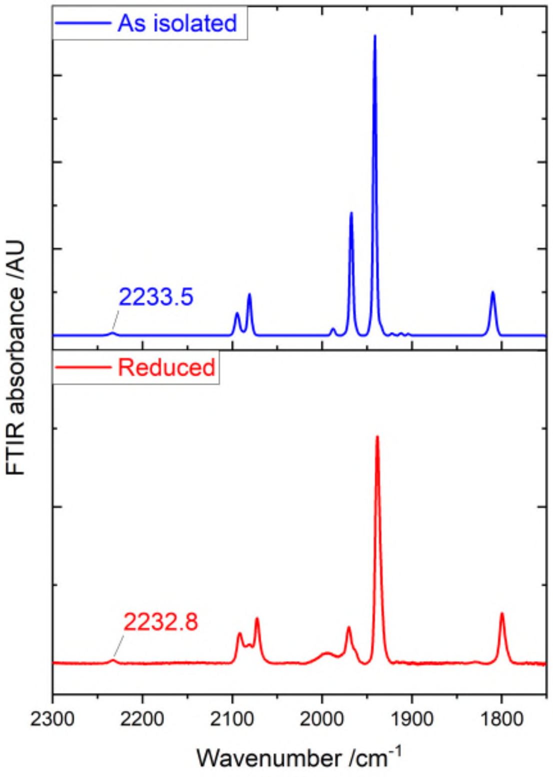

Figure S18. IR spectra of F27CNF *Dd*HydAB^{PDT} in the as-isolated state, and reduced by 10 mM Eu(II)-DTPA (DTPA = diethylenetriamine pentaacetate), in 100 mM Tris-HCl buffer containing 150 mM NaCl, pH8, 1cm^{-1} resolution, 1024 co-averaged scans.

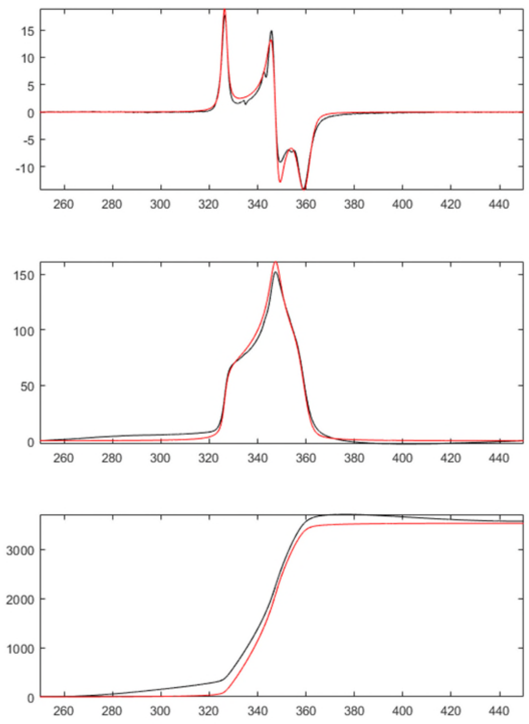

Figure S19. EPR spectrum of 200 uM F27CNF apo-*Dd*HydAB, in pH8 100 mM Tris buffer, temperature at 10K. Total spin integral is 0.15 spins per apo-*Dd*HydAB protein molecule; b. ~one EPR signal: g = [2.0526,1.928,1.864]; lw/MHz = [37 66 121].

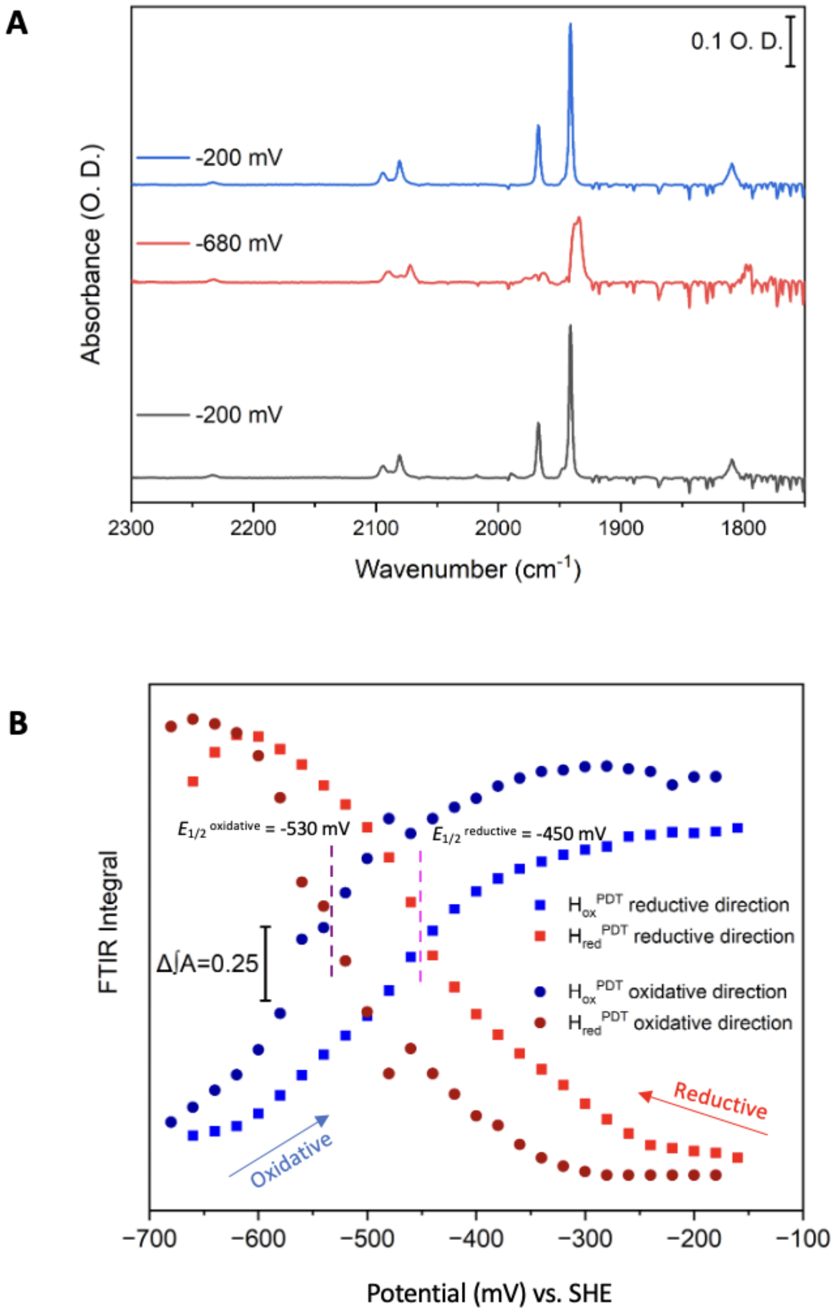

Figure S20. IR Spectroelectrochemistry of F27CNF *Dd*HydAB^{PDT}. A: stack of spectra showing the enzyme poised at -200 mV, then reduced at -680 mV, then re-oxidised at -200 mV, showing full recovery of the original state. B: Potential dependence of populations of different species within the enzyme; light blue and red traces represent spectral integrals measured for different bands during steps towards more negative potential; dark blue and brown traces represent spectral integrals measured for different bands during steps towards more positive potential. The oxidative titration is the same as that shown in Figure 5C of the main text. Some hysteresis is observed.

Table S1. Summary of primers used for the production of FdI proteins produced during this work. Their characteristics are denoted as 'native-like' indicating that the variant was indistinguishable from native FdI' protein in terms of its UV-visible spectroscopic or electrochemical characteristics; 'altered' to indicate non-native-like spectral or redox properties; or 'no protein' to indicate that no protein production was observed for this variant.

Desired FdI protein	Primers used (5'à3')	Description	Characterisation

Native	cacacagaattcattaaagaggagaaattaactatgg	Forwards primer for amplification of FdI gene with an EcoRI site at 5' end	n/a
Native	cggccgctcgagttatgcggtcagttcctcttct	Forwards primer for amplification of FdI gene with an XhoI at 3' end	n/a
Y3F	cccggaattcgcagcaTTCaaagttaccctggtta<br>taaccagggtaactttGAAtgctgcgaattccggg	Forwards and reverse primers for the mutation of tyrosine 3 to phenylalanine	Native-like*
Y3W	cccggaattcgcagcaTGGaaagttaccctggtta<br>taaccagggtaactttCCAtgctgcgaattccggg	Forwards and reverse primers for the mutation of tyrosine 3 to tryptophan	Native-like*
Y3A	cccggaattcgcagcaGCAaaagttaccctggtta<br>taaccagggtaactttTGCtgctgcgaattccggg	Forwards and reverse primers for the mutation of tyrosine 3 to alanine	Native-like*
Y23F	tgtccggatgatgtgTTCattctggatgcagcaga<br>tctgctgcatccagaatGAAcacatcatccggaca	Forwards and reverse primers for the mutation of tyrosine 23 to phenylalanine	Altered
Y23W	tgtccggatgatgtgTGGattctggatgcagcaga<br>tctgctgcatccagaatCCAcacatcatccggaca	Forwards and reverse primers for the mutation of tyrosine 23 to tryptophan	Altered
Y23A	tgtccggatgatgtgGCGattctggatgcagcaga<br>tctgctgcatccagaatCGCcacatcatccggaca	Forwards and reverse primers for the mutation of tyrosine 23 to alanine	Altered
Y37F	ggtattgatctgccgTTTagctgtcgtgcaggt<br>acctgcacgacagctAAAcggcagatcaatacc	Forwards and reverse primers for the mutation of tyrosine 37 to phenylalanine	Native-like
Y37W	ggtattgatctgccgTGGagctgtcgtgcaggt<br>acctgcacgacagctCCAcggcagatcaatacc	Forwards and reverse primers for the mutation of tyrosine 37 to tryptophan	Native-like
Y37A	ggtattgatctgccgGCGagctgtcgtgcaggt<br>acctgcacgacagctCGCcggcagatcaatacc	Forwards and reverse primers for the mutation of tyrosine 37 to alanine	Native-like
Y37CNF	ggtattgatctgccgTAGagctgtcgtgcaggt<br>acctgcacgacagctCTAcggcagatcaatacc	Forwards and reverse primers for the mutation of tyrosine 37 to an amber stop codon for production of cyanophenylalanine-labelled FdI	Native-like
F63Y	caggatgatcagagcTATctggatgatgaccag<br>ctggtcatcatccagATAgctctgatcatcctg	Forwards and reverse primers for the mutation of phenylalanine 63 to tyrosine	Native-like‡
F63W	caggatgatcagagcTGGctggatgatgaccag<br>ctggtcatcatccagCCAgctctgatcatcctg	Forwards and reverse primers for the mutation of phenylalanine 63 to tryptophan	Native-like‡
F63A	caggatgatcagagcGCGctggatgatgaccag<br>ctggtcatcatccagCGCgctctgatcatcctg	Forwards and reverse primers for the mutation of phenylalanine 63 to alanine	Native-like‡
Y80F	tctgacctgtgcagccTTCccggttagtgatgtta<br>taacatcactaaccggGAAggctgcacaggtcaga	Forwards and reverse primers for the mutation of tyrosine 80 to phenylalanine	No protein
Y80W	tctgacctgtgcagccTGGccggttagtgatgtta<br>taacatcactaaccggCCAggctgcacaggtcaga	Forwards and reverse primers for the mutation of tyrosine 80 to tryptophan	No protein
Y80A	tctgacctgtgcagccGCGccggttagtgatgtta<br>taacatcactaaccggCGCggctgcacaggtcaga	Forwards and reverse primers for the mutation of tyrosine 80 to alanine	No protein
FdI_Strep_F	5' accatcctccaaaatcggatctggaagttc	Forward primer for the amplification of whole protein containing amber codon	Full-length protein containing non-native amino acid
FdI_Strep_R1	5' tgtgaccagctaccgccaccaccaccgcttgcggtcagttcct cttctt	First reverse primer for the amplification of whole protein containing amber codon	Full-length protein containing non-native amino acid
FdI_Strep_R2	gccgctcgagttatttttcaaactgcggatgtgaccagctaccg ccacc	Second reverse primer for the amplification of whole protein containing amber codon	Full-length protein containing non-native amino acid

*** Although the Y3 variants show electrochemistry and UV-visible spectroscopy that look similar to the wild type enzyme, the position of residue 3 is at a significant distance (ca 22 Å) from the FeS cluster and so we did not pursue this variant beyond the initial small batch of protein used for this characterisation.**

ǂ We also prepared a variant deliberately truncated at position 63 by incorporation of the amber stop codon but including no CNF in the growth medium. For the truncated protein, absorbance in the visible region around 420 nm suggested some cluster incorporation: residue 63 is far enough through the sequence that three of the four cysteines which normally ligate the 2Fe2S cluster are present in the truncated protein, and we hypothesise that these are sufficient to support some cluster formation. Therefore, although position 63 looked promising in terms of the proximity of this site to the 2Fe2S cluster of Fdx, and in terms of the properties of variants with native amino acid mutations at this position, we were concerned that any truncated protein formed as a side product during expression of the CNF variant at position 63 would contribute to spectroscopic and electrochemical signals, and the similarity in size to the full-length protein would make it difficult to separate. For this reason, we did not continue with CNF incorporation at residue 63.

Table S2. Melting temperature of wild type FdI and variants.

Protein variant	Melting Temperature (duplicate measurements measured in degrees Celsius)
WT	44.0, 44.1
Y3F	42.1, 42.5
Y37F	42.2, 42.3
Y3CNF	43.2, 43.3
Y37CNF	44.8, 44.4

Table S3. X-ray data collection parameters.

Protein variant	Wild Type Fd (As-isolated)	Wild Type Fd (Reduced)	Wild Type Fd (Oxidised)	Fd_Y37CNF (Reduced)	Fd_Y37CNF (Oxidised)	F27CNF apo_*Dd*H	F27CNF *Dd*HydAB^{PDT} (Reduced)	F27CNF *Dd*HydAB^{PDT} (Oxidised)
Beamline	I03	I03	I03	I24	I03	I03	I03	I03
Wavelength	0.77 Å	0.62 Å	0.62 Å	0.97 Å	0.77 Å	0.77 Å	0.81	Å
Detector	Eiger2 XE 16M	Eiger2 XE 16M	Eiger2 XE 16M	Pilatus3 6M	Eiger2 XE 16M	Eiger2 XE 16M	Eiger2 XE 16M	Eiger2 XE 16M
Resolution	0.92 Å	1.0 Å	1.20 Å	1.10 Å	1.10 Å	1.02 Å	0.97 Å	1.02 Å
Data reduction software	Dials	Autoproc	Dials	Dials	Dials	Dials	Dials	Dials
Refinement software	Phenix_refine	Phenix_refine	Refmac5	Phenix_refine	Refmac5	Phenix_refine	Phenix_refine	Phenix_refine

X-ray data collection and refinement statistics

Table S4. X-ray data collection and refinement statistics for Ferredoxin variants

PDB code	9GYD	9GYN	9GYL	9GYU	6GYR
Ferredoxin variant	Wild Type (As-isolated)	Wild Type (Reduced)	Wild Type (Oxidised)	Y37CNF (Reduced)	Y37CNF (Oxidised)
Space group	$P2_12_12_1$	$P2_12_12_1$	$P4_32_12$	$P2_12_12_1$	$P2_1$
Unit cell dimensions (Å)	a=30.30, b=51.41, c=61.33	a=30.29, b=51.29, c=61.25	a=62.05, b=62.05, c=85.57	a=28.89, b=46.72, c=63.33	a=58.84, b=61.53, c=58.84 β=119.24
Resolution (Å)	39.4-0.92 (0.94-0.92)	39.3-1.00 (1.03-1.00)	50.2-1.20 (1.22-1.20)	53.24-1.10 (1.12-1.10)	39.43-1.10 (1.12-1.10)
Total reflections	835,290 (33,211)	1,276,735 (39,441)	1,374,029 (63,822)	633,572 (15,345)	966,853 (47,869)
Unique reflections	67,214 (3,289)	52,421 (2,565)	54,217 (2,631)	33,332 (1,372)	148,067 (7,311)
Completeness (%)	100 (100)	100 (100)	100 (99.7)	93.9 (78.4)	100 (100)
Multiplicity	12.4 (10.1)	24.4 (15.4)	25.3 (24.3)	19.0 (11.2)	6.5 (6.5)
<I/s>	18.2 (0.8)	15.7 (1.1)	14.8 (0.5)	15.2 (1.5)	11.0 (1.8)
R_{merge}	0.049 (1.19)	0.081 (2.5)	0.10 (3.2)	0.098 (1.6)	0.064 (0.804)
R_{pim}	0.014 (0.37)	0.017 (0.66)	0.02 (0.93)	0.022 (0.49)	0.029 (0.376)
$CC_{1/2}$	1.0 (0.8)	0.999 (0.672)	1.0 (0.378)	0.999 (0.654)	0.997 (0.583)
Refinement					
R_{work}/R_{free} (%)	12.6/13.6	14.1/15.5	15.5/17.8	16.2/17.3	13.1/14.4
No. of atoms	940	941	922	968	3858
Macromolecule	793	790	783	830	3118
Solvent	147	151	139	138	740
Average B-factors					
Macromolecule	16.9	16.9	18.9	13.5	14.5
Solvent	29.9	28.3	29.3	22.0	24.8
RMSD bond lengths (Å)	0.013	0.019	0.016	0.008	0.004
RMSD angles (°)	1.45	2.3	2.3	1.04	0.75
Ramachandran plot favoured / outliers	99/0	99/0	97.9/0	99/0	99.2/0

Clashscore (percentile)	1.95 (95th)	1.96 (97th)	0.66 (99th)	0.62 (99th)	2.18 (97th)
Molprobity score (percentile)	0.96 (98th)	0.99 (98th)	0.77 (100th)	0.71 (99th)	0.99 (98th)

Values in parenthesis refer to highest resolution shell

Table S5. X-ray data collection and refinement statistics for F27CNF *Dd*HydAB.

PDB code	9GZL	9GZ4	9GZ0
DdH variant	F27CNF apo-*Dd*HydAB	F27CNF *Dd*HydAB^{PDT} (Reduced)	F27CNF *Dd*HydAB^{PDT} (Oxidised)
Space group	$P2_12_12_1$	$P2_12_12_1$	$P2_12_12_1$
Unit cell dimensions (Å)	a=50.46, b=88.01, c=92.86	a=49.72, b=87.71, c=89.89	a=49.73, b=87.83, c=89.86
Resolution (Å)	63.9-1.02 (1.04-1.02)	62.8-0.96 (0.98-0.96)	62.8-1.02 (1.04-1.02)
Total reflections	2,714,291 (80,193)	3,055,711 (120,258)	5,339,550 (261,150)
Unique reflections	209,983 (10,197)	239,126 (11,725)	200,276 (9,779)
Completeness (%)	99.9 (97.5)	100 (99.9)	99.9 (98.3)
Multiplicity	12.9 (7.9)	12.8 (10.3)	26.7 (26.7)
<I/s>	10.9 (0.3)	10.8 (0.9)	12.5 (0.5)
R_{merge}	0.095 (2.7)	0.107 (2.2)	0.13 (3.6)
R_{pim}	0.027 (0.99)	0.032 (0.74)	0.026 (0.71)
$CC_{1/2}$	1.0 (0.3)	0.999 (0.470)	0.999 (0.377)
Refinement			
R_{work}/R_{free} (%)	13.9/15.3	14.4/15.2	14.2/16.6
No. of atoms	4430	4464	4176
Macromolecule	3842	3938	3960
Solvent	588	526	535
Average B-factors			

Macromolecule	17.9	14.2	16.7
Solvent	26.2	25.9	28.4
RMSD bond lengths (Å)	0.009	0.008	0.012
RMSD angles (°)	1.1	1.2	1.3
Ramachandran plot favoured / outliers	97.9/0	97.7/0	97.7/0
Clashscore (percentile)	4.2 (80th)	3.44 (84th)	2.37 (95th)
Molprobity score (percentile)	1.23 (90th)	1.20 (91st)	1.09 (96th)

Values in parenthesis refer to highest resolution shell

References

[1] [a]M. Toplak, G. Birarda, S. Read, C. Sandt, S. M. Rosendahl, L. Vaccari, J. Demšar, F. Borondics, *Synchrotron Radiation News* **2017**, *30*, 40-45; [b]M. Toplak, S. T. Read, C. Sandt, F. Borondics, *Cells* **2021**, *10*.
[2] J. T. Reilly, J. M. Walsh, M. L. Greenfield, M. D. Donohue, *Spectrochim Acta A* **1992**, *48*, 1459-1479.
[3] [a]G. Winter, D. G. Waterman, J. M. Parkhurst, A. S. Brewster, R. J. Gildea, M. Gerstel, L. Fuentes-Montero, M. Vollmar, T. Michels-Clark, I. D. Young, N. K. Sauter, G. Evans, *Acta Crystallogr D Struct Biol* **2018**, *74*, 85-97; [b]J. Beilsten-Edmands, G. Winter, R. Gildea, J. Parkhurst, D. Waterman, G. Evans, *Acta Crystallogr D Struct Biol* **2020**, *76*, 385-399.
[4] C. Vonrhein, C. Flensburg, P. Keller, A. Sharff, O. Smart, W. Paciorek, T. Womack, G. Bricogne, *Acta Crystallographica Section D: Biological Crystallography* **2011**, *67*, 293-302.
[5] A. J. McCoy, R. W. Grosse-Kunstleve, P. D. Adams, M. D. Winn, L. C. Storoni, R. J. Read, *J Appl Crystallogr* **2007**, *40*, 658-674.
[6] P. Emsley, B. Lohkamp, W. G. Scott, K. Cowtan, *Acta Crystallographica Section D: Biological Crystallography* **2010**, *66*, 486-501.
[7] G. N. Murshudov, A. A. Vagin, E. J. Dodson, *Acta Crystallographica Section D* **1997**, *53*, 240-255.
[8] D. Liebschner, P. V. Afonine, M. L. Baker, G. Bunkoczi, V. B. Chen, T. I. Croll, B. Hintze, L.-W. Hung, S. Jain, A. J. McCoy, N. W. Moriarty, R. D. Oeffner, B. K. Poon, M. G. Prisant, R. J. Read, J. S. Richardson, D. C. Richardson, M. D. Sammito, O. V. Sobolev, D. H. Stockwell, T. C. Terwilliger, A. G. Urzhumtsev, L. L. Videau, C. J. Williams, P. D. Adams, *Acta Crystallographica Section D* **2019**, *75*, 861-877.
[9] C. J. Williams, J. J. Headd, N. W. Moriarty, M. G. Prisant, L. L. Videau, L. N. Deis, V. Verma, D. A. Keedy, B. J. Hintze, V. B. Chen, S. Jain, S. M. Lewis, W. B. Arendall III, J. Snoeyink, P. D. Adams, S. C. Lovell, J. S. Richardson, D. C. Richardson, *Protein Science* **2018**, *27*, 293-315.
[10] C. Binda, A. Coda, A. Aliverti, G. Zanetti, A. Mattevi, *Acta Crystallographica Section D* **1998**, *54*, 1353-1358.
[11] H. Ohno, K. Takeda, S. Niwa, T. Tsujinaka, Y. Hanazono, Y. Hirano, K. Miki, *Plos one* **2017**, *12*, e0178183.
[12] P. Rodriguez-Macia, K. Pawlak, O. Rudiger, E. J. Reijerse, W. Lubitz, J. A. Birrell, *J Am Chem Soc* **2017**, *139*, 15122-15134.
[13] S. Šašić, A. Muszynski, Y. Ozaki, *The Journal of Physical Chemistry A* **2000**, *104*, 6380-6387.
[14] [a]P. A. Ash, H. A. Reeve, J. Quinson, R. Hidalgo, T. Zhu, I. J. McPherson, M. W. Chung, A. J. Healy, S. Nayak, T. H. Lonsdale, K. Wehbe, C. S. Kelley, M. D. Frogley, G. Cinque, K. A. Vincent, *Anal Chem* **2016**, *88*, 6666-6671; [b]P. A. Ash, R. Hidalgo, K. A. Vincent, *J Vis Exp* **2017**.
